# Paleoclimate-induced stress on polar forested ecosystems prior to the Permian–Triassic mass extinction

**DOI:** 10.1038/s41598-022-12842-w

**Published:** 2022-05-24

**Authors:** Erik L. Gulbranson, Morgan M. Mellum, Valentina Corti, Aidan Dahlseid, Brian A. Atkinson, Patricia E. Ryberg, Gianluca Cornamusini

**Affiliations:** 1 Department of Geology, Gustavus Adolphus College, 800 W. College Ave, St. Peter, MN 56082 USA; 2 Dipartimento di Scienze Fisiche, della Terra e dell’Ambiente, Università di Siena, 53100 Siena, Italy; 3 Department of Ecology and Evolutionary Biology, University of Kansas, 6012 Haworth Hall, Lawrence, KS 66045 USA; 4 Department of Physical and Natural Sciences, Park University, Parkville, MO 64152 USA

**Keywords:** Palaeoclimate, Palaeoecology

## Abstract

The end-Permian extinction (EPE) has been considered to be contemporaneous on land and in the oceans. However, re-examined floristic records and new radiometric ages from Gondwana indicate a nuanced terrestrial ecosystem response to EPE global change. Paleosol geochemistry and climate simulations indicate paleoclimate change likely caused the demise of the widespread glossopterid ecosystems in Gondwana. Here, we evaluate the climate response of plants to the EPE via dendrochronology snapshots to produce annual-resolution records of tree-ring growth for a succession of late Permian and early Middle Triassic fossil forests from Antarctica. Paleosol geochemistry indicates a shift in paleoclimate towards more humid conditions in the Early and early Middle Triassic relative to the late Permian. Paleosol morphology, however, supports inferences of a lack of forested ecosystems in the Early Triassic. The plant responses to this paleoclimate change were accompanied by enhanced stress during the latest Permian as determined by high-resolution paleoclimate analysis of wood growth intervals. These results suggest that paleoclimate change during the late Permian exerted significant stress on high-latitude forests, consistent with the hypothesis that climate change was likely the primary driver of the extinction of the glossopterid ecosystems.

## Introduction

The end-Permian extinction (EPE) was one of the most severe mass extinctions in the history of metazoan life. The effects of the EPE were pronounced for marine organisms, including a nearly instantaneous (~ 30 kyr) record of extinction in paleotropical seaways^1^ that was coincident with a drastic reorganization of seawater chemistry and circulation^2^. It is surmised that terrestrial organisms underwent a similar and synchronous extinction^3^, however, terrestrial flora and fauna during the late Permian and the EPE display localized and asynchronous extinction and origination rates^4–8^. Of the plant lineages that did go extinct in the late Paleozoic–early Mesozoic, the most widespread were the glossopterids of Gondwana.

During the late Permian, glossopterids occurred on every continental landmass of Gondwana, and were the predominant arborescent taxa of terrestrial ecosystems at paleopolar latitudes^9^. These plants were adapted for a broad range of climate conditions, changes of climate state (e.g., icehouse to greenhouse), and persisted as a low-biodiversity floral province throughout the Permian^10^. It is, therefore, a paradox that such a widespread flora was unable to cope with global change during the EPE. In Australia^4^, glossopterids went extinct approximately 370 kyr prior to the marine EPE at 251.939 ± 0.031 Ma^1^, indicating that the specific mechanism(s) to stress these ecosystems differed in type, timing, or magnitude from the marine extinctions. How this array of climate and environmental change directly affected terrestrial ecosystems is a key area of research, but what is known is that ecosystem recovery was delayed, and taxa that filled-in ecosystem niches following the EPE were most likely affected by repeated environmental/climatic stress during the Early Triassic^11,12^.

Elements of the succeeding flora appear dispersed through the Early Triassic and suggest a delayed recovery of paleo-equatorial ecosystems^12^. In Antarctica, however, there is a limited palynofloral or megafloral record of the Early Triassic, with putative leptosporangiate fern spores and megafloral fragments reported from the Allan Hills^13^. Early Middle Triassic megafloras, however, occur across Gondwana^5,14^. In Antarctica, the early Middle Triassic flora was highly diverse in comparison to the Permian counterpart^15^ including: peltasperms, ginkgophytes, corystosperms, conifers, and a higher diversity of understory vegetation (possibly including peltasperms). Depositional systems and soil-forming environments during the Middle Triassic were unlike those of the late Permian^16,17^. In Antarctica, poorly developed soils that were likely waterlogged and wetlands that characterize the late Permian^18^ were succeeded by incipient soil development on non-wetland floodplains in the early Triassic^16^. By the Middle Triassic, deeply weathered soils rich in soil-formed clay and oxide minerals occur coincident with plant megafloral preservation^16,17^.

**Figure 1:**
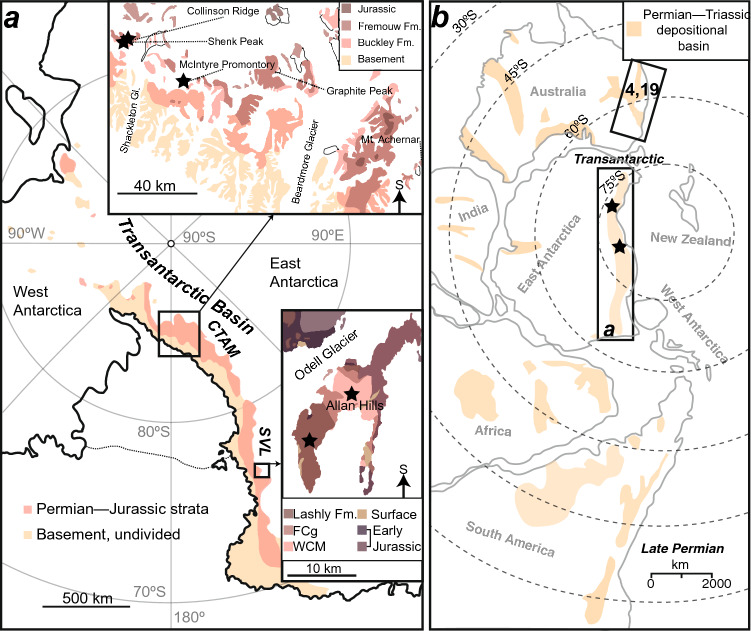
Map and paleogeography of the study area. (**a**) Map of the study locations, CTAM and SVL, on Antarctica. Exposures of major geologic units are shown, ice/snow where not shaded. (**b**) Paleogeographic reconstruction of the late Permian for Gondwana. Permian–Triassic depositional basins are shaded, study locations indicated by stars. The boxed region of eastern Australia highlights the correlative stratigraphy discussed in the text^4,19^. Figure created using Adobe Illustrator, v. 25.0.1, adobe.com.

What were the factors that led to the demise of the glossopterid ecosystems? Recent paleoclimate simulations and sediment geochemistry from eastern Australia postulate a climate-forced stress on plant growth via paleoclimate simulations for the Bowen and Sydney basins^4,14,19^. Here, we test this hypothesis using dendrochronologic analysis of permineralized wood from the Shackleton Glacier area and Southern Victoria Land, Antarctica (Fig. 1). Both regions preserve paleopolar forested ecosystems in the late Permian and early Middle Triassic. It is demonstrated here that just prior to the demise of the glossopterid floral province, a distinct change in decadal-scale paleoclimate oscillations had occurred, which resulted in greater stress on arborescent taxa. Long-term analysis of paleoprecipitation and evapotranspiration from paleosols in the study region contextualize these stresses as being primarily related to increasing ratios of precipitation to evapotranspiration, concurrent with climate warming. While the paleoclimate in the early Middle Triassic was similar to the latest Permian, the plant responses to this climate were markedly more amenable in the Triassic than in the latest Permian. This result stands in stark contrast to the earlier late Permian record of plant-climate response, suggesting that by the latest Permian the polar forested ecosystems were in a state of disequilibrium, with plants failing to adapt to a sharply-changing climate.

## Geologic setting and age

**Figure 2:**
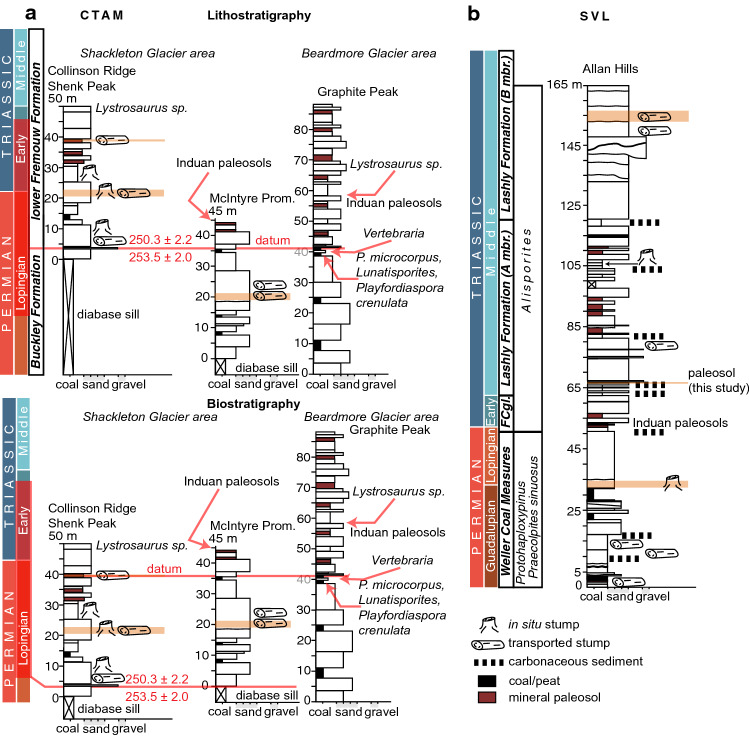
Stratigraphy of the study area. (**a**) Permian and Triassic stratigraphy of the central Transantarctic Mountains (CTAM). Measured sections from Collinson Ridge, McIntyre Promontory, and Graphite Peak are displayed in two configurations. The lithostratigraphy configuration places a datum at the contact between the Buckley and Fremouw formations. The available maximum depositional ages^21^ for these strata are indicated and are derived from Collinson Ridge and Layman Peak. The orange highlighted areas denote the fossil wood used in this study. Palynology^20^ is shown for Graphite Peak as it produces regionally significant pollen near the lithostratigraphic contact. The biostratigraphy configuration places the datum at the last appearance of coal or glossopterid megafossils. The red bar on the timescale refers to the ranges of the maximum depositional ages plus their uncertainties. (**b**) Permian and Triassic stratigraphy for southern Victoria Land (SVL), Allan Hills (after^17^). FCgl. refers to the Feather Conglomerate. The available palynology is indicated next to the timescale. The lowest most Triassic paleosol indicative of plant productivity used in this study is indicated along with stratigraphic positions of fossil wood used in this study. Figure created using Adobe Illustrator, v. 25.0.1, adobe.com.

Antarctica hosted several depositional basins that were actively subsiding during the late Paleozoic and early Mesozoic, of these the Transantarctic Basin was the largest (Fig. 1a). During the Permian–Triassic the Transantarctic Basin was situated close to the paleo south pole (Fig. 1b). The foreland-style basin preserves predominantly terrestrial strata with abundant plant fossils (Fig. 2). Stratigraphic names and correlations are broadly subdivided by their occurrence in the central Transantarctic Mountains (CTAM) area and southern Victoria Land (SVL, Fig. 2a,b). In the CTAM succession, Permian strata include the Buckley Formation and part of the lower Fremouw Formation in the vicinity of the Shackleton Glacier area^20,21^. The lower Fremouw Formation is recognized as diachronous based on the presence of glossopterid megafloral remains in these lithofacies^20^ and based on maximum depositional ages that span the latest Permian–Early Triassic^21^. Triassic strata in the CTAM area include the remainder of the Fremouw Formation, and the Falla Formation in the SVL succession, Permian strata include the Weller Coal Measures, whereas Triassic strata include the Feather Conglomerate and Lashly Formation.

In the CTAM area, palynology, vertebrate biostratigraphy, and maximum depositional ages based on U–Pb analyses on zircon crystals constrains the age information for these successions. *Protohaploxypinus microcorpus* has been recovered from the upper Buckley Formation at Graphite Peak below the first occurrence of *Lystrosaurus* (Fig. 2a)^20^. This pollen association was used to define a Changhsingian age for these strata^20^ prior to the more recent revision of the *P. microcorpus* Zone in eastern Australian. In the Shackleton Glacier area (Layman Peak), a maximum depositional age of 253.5 ± 2.0 Ma from U–Pb analyses on zircon confirms a late Permian age of the Buckley Formation However, at Collinson Ridge, the contact of the Buckley and Fremouw formations has yielded a maximum depositional age of 250.3 ± 2.2 Ma (Fig. 2a)^21^. Moreover, *P. microcorpus* occurs in the upper Buckley Formation at Graphite Peak in association with *Vertebraria*. Thus, there are two significantly different paleobiologic scenarios in these strata that depend on how the available chronologic data are organized (Fig. 2a). If it is assumed that the lithostratigraphic separation of the Buckley and Fremouw formations is not diachronous, then the choice of the Buckley and Fremouw contact as a datum result in: (1) an older age for *P. microcorpus* in Antarctica relative to the Induan age of *P. microcorpus* Zone in eastern Australia^14^, however, the first occurrence of *P. microcorpus* is often in the Lopingian; and (2) possible extension of glossopterid ecosystems in the Transantarctic Basin in the vicinity of Collinson Ridge to much younger time intervals in the late Permian or possibly the Early Triassic. Conversely, if the last appearance of coal and/or glossopterid megafossils is used as a datum in the CTAM area, the implications are: (1) synchronous disappearance of glossopterid megafossils over a time range that is consistent with eastern Australia but with the possibility of a much younger time for the demise of glossopterids in Antarctica; and (2) a more consistent timing of the first occurrence of *P. microcorpus* in Antarctica with the *P. microcorpus* Zone eastern Australia. However, the potential for floral provincialism across Gondwana^22^ does leave open the possibility that *P. microcorpus* in Antarctica is evidence of range expansion of the plants that produced this pollen. In both cases, however, the demise of glossopterids on Antarctica is either consistent with or younger than the high-precision calibration of glossopterid extinction in eastern Australia^4^ with the potential that the paleo high-latitudes acted as refugia for these plants during late Permian global change^4,14,19^.

Late Permian fossil wood was collected from the upper Buckley Formation at McIntyre Promontory from a dense interval of allochthonous fossil wood ~ 15 m from the uppermost carbonaceous shale in the succession (Fig. 2a). Fossil wood with glossopterid affinities and co-occurring with *Vertebraria* at Shenk Peak and Collinson Ridge were collected in the lower Fremouw Formation at 10–30 m, respectively, below the first occurrence of *Lystrosaurus* remains (Fig. 2a). The Shenk Peak fossil wood occurs as sub horizontal to horizontal wood fragments in the upper horizon of a densely *Vertebraria*-rooted paleosol. The Collinson Ridge material occurs as predominantly in situ fossil stumps, with a few samples of allochthonous fossil wood. The middle Fremouw Formation, and lower Fremouw Formation in the Beardmore Glacier area, contain the palynomorph *Aratisporites parvispinosus*, which corresponds to the late Early Triassic *Protohaploxypinus samoilovichii* and *Aratisporites tenuispinosus* biozones of eastern Australia^14^. The upper Fremouw Formation contains fossils of *Angonisaurus*, which has been correlated to the *Cynognathus* Assemblage Zone, *Criodon-Ufudocyclops* subzone of the Karoo Basin^23^, indicating an Anisian age for the upper Fremouw Formation based on the age range of this subzone^24^. The vertebrate biostratigraphy and palynology all suggest a late Early (Olenekian) to early Middle (Anisian) Triassic age for the Fremouw Formation outside of the Shackleton Glacier area.

In SVL, palynology and relative dating are the principal information available for age determination. The Weller Coal Measures yield pollen and spore assemblages of the *Protohaploxypinus* Biozone and *Praecolpites sinuosus* consistent with a Guadalupian to Lopingian age^25^ (Fig. 2b). The contact between the Feather Conglomerate and Weller Coal Measures has been interpreted as conformable^16^, but may be locally disconformable elsewhere in the depositional basin. The Feather Conglomerate and the Lashly A and B members yield palynomorphs of *Alisporites*^26^ of the *Protohaploxypinus samoilovichii* and *Aratisporites tenuispinosus* biozones of eastern Australia, indicating a late Early Triassic age^14^ (Fig. 2b). Fossil wood with glossopterid affinities was collected from the upper portion of the Weller Coal Measures as a mixture of in situ stump samples and allochthonous wood samples. Fossil wood with affinities to corystosperms, and associated with *Dicroidium* leaves, were collected from the Lashly B member in the Allan Hills as allochthonous wood samples.

## Results

### Dendrochronology

**Figure 3:**
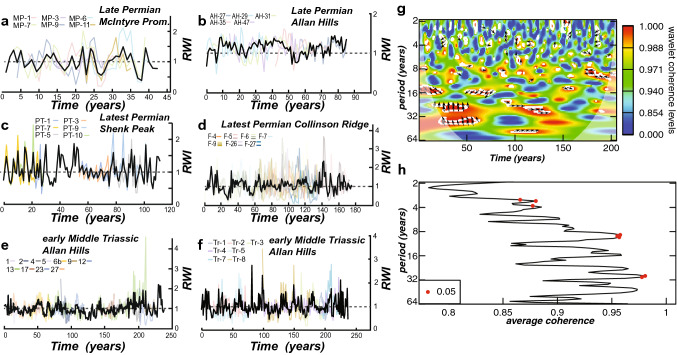
Ring width index (RWI) and correlation of the replicate early Middle Triassic chronologies (**g**–**h**). (**a**–**f**), RWI plots of cross-matched tree ring width data. (**g**) Wavelet scalogram of wavelet coherence between the two replicate Triassic chronologies: color spectrum is equivalent to the correlation of the two chronologies (0–1); the arrows indicate positive (pointing right) or negative correlation (pointing left); the up/down direction of arrows indicates if one chronology leads/lags another, respectively (irrelevant for this analysis as the two chronologies are not cross-matched to each other); white lines indicate the 0.05 significance level of wavelet coherence. (**h**) Fourier period versus average wavelet coherence, the red dots signify Fourier periods significant at the 0.05 level for the specified wavelet correlation between the two chronologies. Figure created using Adobe Illustrator, v. 25.0.1, adobe.com.

**Figure 4:**
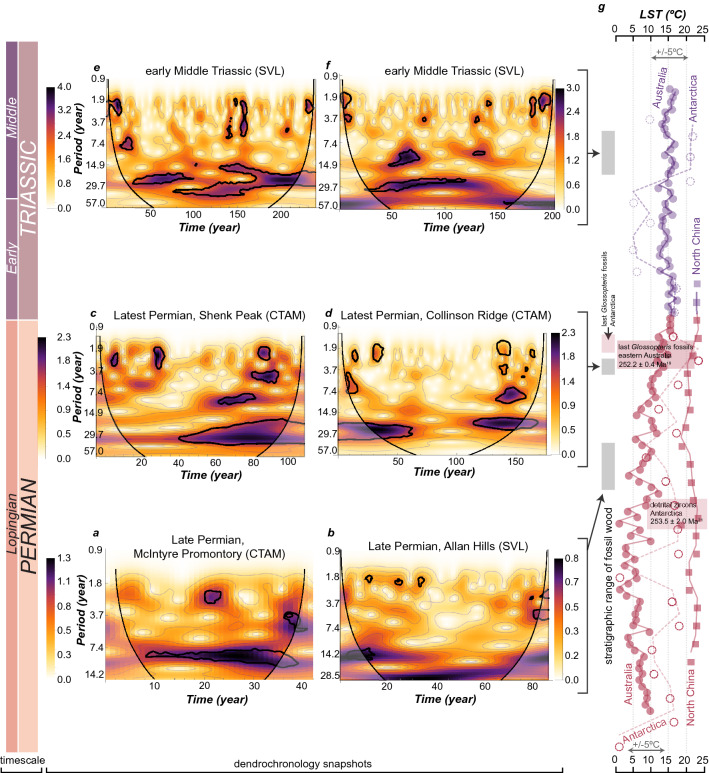
Wavelet scalograms of the RWI data and land surface temperature (LST). (**a**–**f**) The color spectrum indicates wavelet power (square of wavelet coefficient), higher power indicating a stronger signal in the data. The shaded envelope is the cone of influence reflecting wavelet coefficients that are erroneous near the edge of each time series. The dark lines indicate the wavelet power domains that are significant as compared to red noise at the 0.05 level. The abscissa represents the time represented in each chronology and is directly related to the RWI data. The ordinate axis represents the Fourier period and is scaled with 16 voices per octave. (**g**) Land surface temperature (LST) through time in Antarctica (dashed line and dashed circles)^18^, eastern Australia^19^ (solid line and filled circles), and North China^28^ (solid line and filled squares). The symbols indicate average values binned evenly through each time interval, the lines are running averages of the data. Purple colors indicate Triassic data, and red colors indicate Permian data. LST estimates are based on the chemical index of alteration (CIA)^29^ and are corrected for authigenic K concentrations^19,30^. Data for eastern Australia represent sedimentary CIA values, and are averaged between the Bowen and Sydney basins based on existing correlations and U–Pb ages^19^. Data for Antarctica is from Graphite Peak and represent paleosol CIA values^18^. Markers indicating the last occurrence *Glossopteris* fossils are shown for Antarctica^20^ and eastern Australia^4,19^. The detrital zircon U–Pb for Antarctica is from Layman Peak in the Shackleton Glacier area^21^. Figure created using Adobe Illustrator, v. 25.0.1, adobe.com.

**Figure 5:**
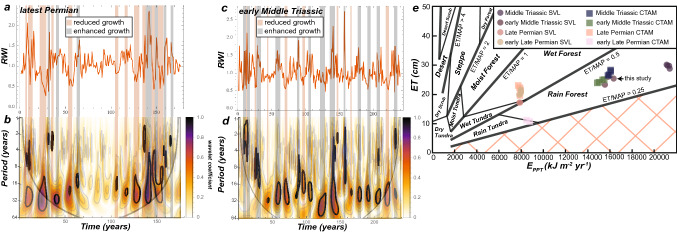
Tree growth patterns and paleoclimate. (**a**,**b**) RWI and CWT analysis with the derivative of a gaussian (DOG, 2nd derivative) wavelet for the Late Permian Collinson Ridge chronology. (**c**,**d**) RWI and CWT analysis via the DOG wavelet for the Triassic Allan Hills chronology. Intervals of reduced/enhanced growth as determined by RWI that correlate with significant wavelet coefficients are illustrated by the shaded vertical bars. (**e**) Paleoclimate model results from paleosol geochemistry at each area in the study region from the Permian–Triassic, ET = evapotranspiration, Eppt = energy from precipitation, where these parameters are calculated from the equations for paleosol-based paleoclimate proxies^31,32^. Humidity provinces and floral regimes fall between each of the lines, and the calculated ET and Eppt values from each paleosol are shown by the symbols. Figure created using Adobe Illustrator, v. 25.0.1, adobe.com.

Cross-matched tree-ring widths (TRW) are converted to an index called the Ring Width Index (RWI), which evaluates the measured TRW in the sample against the expected TRW produced from a spline fitted to the data (Fig. 3a–f). Climate, being one of the state factors for tree growth, is anticipated to be a maximum signal in these RWI values due to the principle of ecologic amplitude^27^, where these trees grew at the limit of their natural range. To detect these signals in RWI time series, we apply the technique of continuous wavelet transform (CWT) analysis to this data. One of the key visualizations of CWT results is in the form of a wavelet scalogram (Figs. 3g, 4 and 5), which is a visualization of the Fourier period on the ordinate axis and the time dimension on the abscissa. The color map in the scalogram refers to the power (square of the wavelet coefficient) of the wavelet against the time series. The null hypothesis for this analysis is of a red noise spectrum, and regions at 0.05 significance level against the null hypothesis are illustrated by bold dark lines. The conical feature, determined by the e-folding time of the wavelet, in each scalogram reflects the region where edge-effects create spurious correlations. Replication of these dendrochronologic results is assessed by statistical comparison of two nearly identical TRW chronologies from the Lower Triassic Lashly Formation using a cross-wavelet analysis (Figs. 3g,h). Statistics of inter-tree cross-matches are provided in Supplementary file 1 and are organized by the location of the samples used to develop each TRW chronology. As a supplement to this table the: (1) the chronology length; (2) subsample signal strength; and (3) distribution of anomalously wide/narrow rings are reported below.

The stratigraphically lowest samples reported here are from the upper Buckley Formation, McIntyre Promontory (see supplementary file 1 for more detail). Six cross-matched samples produce a chronology of 42 years. This chronology is ~ 2 log_2_ units less than the other data sets reported here, which indicates it has a shorter range of scales than the other chronologies (Fig. 3a). Thus, the decreased sample length inhibits comparisons of the longer periodicities extracted from the other chronologies reported here. However, the chronology is robust with a mean overlap of individual TRW records of 20 years. The mean correlation between radii of individual trees (intra-tree correlation) is better than 0.7, with percent parallel covariation better than 80%. For paleoclimate inference, we use the subsample signal strength (SSS), which is a measure of the depth of information contained in a chronology based on the number of overlapping measured transects, the number of trees represented, and the interseries correlation between trees. An arbitrary cutoff of 0.5 is used by convention to assess chronology lengths that are suitable for paleoclimate analysis (SSS > 0.5). The SSS cutoff of 0.5 is reached by year 22 of the 42 years chronology. By comparison, the TRW chronology from the Weller Coal Measures, Allan Hills, exceeded the SSS cutoff by year 26 of the 86 years chronology (Fig. 3b). For the McIntyre Promontory chronology and the Weller Coal Measures, very narrow rings are well-correlated throughout the chronology, whereas anomalously wide rings correlate well, but are not well-expressed in every sample. Although not precisely correlated across the Transantarctic Basin, these fossil wood chronologies are both approximately 15 m below the top of the respective lithostratigraphic unit contacts between upper Permian and lower Triassic strata.

Individual fossil trees from Shenk Peak are associated with an intensely root-turbated paleosol preserving vertical to subvertical *Vertebraria* fossils. Fossil wood from Shenk Peak is stratigraphically higher than the samples from McIntyre Promontory, occurring within the lithostratigraphic division of the lower Fremouw Formation^20^ Of the seven samples measured, one did not produce a reliable cross-match due to limited overlap at either end of the chronology. The remaining six samples produce a chronology of 113 years. This chronology is within log_2_ dimensions of the: Allan Hills (late Permian), Collinson Ridge (latest Permian), and Triassic chronologies; thus, producing meaningful comparisons of periodicity of RWI variation of these four TRW records (Fig. 3c). The mean correlation between radii of individual trees (intra-tree correlation) is better than 0.8, with percent parallel covariation better than 85%. The SSS cutoff of 0.5 is reached by year 35 of the 113 years chronology. Very narrow rings that occur are exceptionally well-correlated with the exception of sample five at year 27 of the chronology. Unlike the stratigraphically lower chronologies, wide rings are well-correlated and expressed well in each sample.

Individual fossil trees from Collinson Ridge were sampled in the field as part of one of three in situ fossil forests in the lower Fremouw Formation The sampled fossil forest contains at least 27 in situ fossil trees interbedded with air-fall tuff and siltstones containing *Glossopteris* compressions/impressions and *Vertebraria* fossils. Seven measured samples produce a chronology of 176 years. The mean correlation between radii of individual trees (intra-tree correlation) is better than 0.75, with percent parallel covariation better than 80% (Fig. 3d). The SSS cutoff value of 0.5 is reached by year 55 of the 176 years chronology, indicating a significant portion of the chronology preserves climate signals. Very narrow rings are well-correlated, and wide rings are similarly correlated and expressed between different samples.

Triassic fossil wood was cross-matched from the Lashly B member, Allan Hills, SVL. The results of these cross-matches are reported elsewhere^17^. This data is used herein to make geospatial comparisons of late Permian TRW chronologies in between the CTAM and SVL regions of Antarctica and for temporal comparisons from the late Permian through the early Middle Triassic. Triassic fossil wood from the Allan Hills resulted in a 238 years chronology exclusively from allochthonous fossil wood. Triassic fossil wood reaches the 0.5 SSS cutoff at year 30 of the 238 years chronology, yielding a long-lived record of paleoclimate history (Fig. 3e). Narrow rings are well-correlated, and wide rings are correlated with the exception of sample one between years 25–30 of the chronology.

### Reproducibility of dendrochronologic results

The cross-wavelet power spectrum^33^ applied to the two independently sampled and measured Triassic TRW chronologies (by authors ELG and VC, Fig. 3e,f) produces significant power (0.05 significance level) for Fourier periods ranging from 14–16 years and 20–64 years, with negative correlations existing for Fourier periods 14–16 years and positive correlations for Fourier periods 16 years and 32 years (Fig. 3g). Wavelet coherence, analogous to a correlation coefficient ranging from 0 to 1, is significant at the 0.05 level for Fourier periods 3–4 years, 10 years, and 32 years (Fig. 3h). Average coherence values are better than 0.85 for the higher frequency signals, and better than 0.95 for the lower frequency signals. The phase is complex for higher frequencies, displaying both lag/lead patterns and positive/negative correlations with respect to time. However, the phase is more organized at lower frequencies with either the ELG chronology lagging behind the VC chronology, or positive correlation between the two chronologies. The range of Fourier periods with significant cross-wavelet power and wavelet coherence are identical to the significant Fourier periods identified in each chronology individually using CWT analysis (Fig. 4e,f), with 0.95 average wavelet coherence for the prominent lower frequency signals in both TRW chronologies.

### Continuous wavelet transform

CWT results for the lowest stratigraphic position of late Permian fossil wood (upper Buckley Formation, McIntyre Promontory; Weller Coal Measures, Allan Hills) indicate a lack of Fourier periods > 20 years, with significant signals at the 2 years, 3–5 years, and 9–15 years (Fig. 4a,b). The higher frequency signals are not consistent over the length of the chronology, however, the shorter frequency signals are more consistent. For the stratigraphically highest late Permian fossil wood samples (lower Fremouw Formation, Shenk Peak; Collinson Ridge) there is a similar range of high frequency signals as for the stratigraphically lower samples, however, there is an emergence of prominent Fourier periods in the 20–30 years range that are continuous or more frequently occurring throughout a chronology (Fig. 4c,d). The Collinson Ridge chronology displays a marked lack of significant high frequency signals for nearly a century, with high frequency signals occurring over ~ 50 years durations on either end of the chronology. The Triassic chronologies (Allan Hills) display intermittent high frequency signals and more persistent periodicities in the 15–30 years range, with potentially minor contributions of periods in the ~ 50 years range (Fig. 4e,f).

### Geochemistry

Long-term paleoclimate averages from the sedimentary record are developed here through upper Permian to lower and middle Triassic strata. Comparisons of reconstructed paleoclimates are made between the well-studied Sydney and Bowen basins^4,19,34^ and the study area of the Transantarctic Basin^18^. Sediment geochemistry data from the Bowen and Sydney basins^19,34^ is correlated between several cores, binned by the number of samples per meter in the cores, and averaged to produce a summary of temporal trends in chemical weathering. The paleo-land surface temperature (LST) is reconstructed via linear relationship with the chemical index of alteration (Fig. 4g). Paleosol geochemistry from Graphite Peak, Transantarctic Basin^18^ was converted to the K-corrected chemical index of alteration and LSTs were estimated from this data (Fig. 4g). The two stratigraphic records of LSTs produce differing magnitudes of variation over time, with the Sydney and Bowen basins preserving a more gradual change per unit time, but with clear oscillations (Fig. 4g). In contrast, the paleosol results from Graphite Peak display large variations in LST estimates. However, both study areas produce similar long-term trends in paleo-LST, with: (1) a > 10 °C warming during the late Permian; and (2) identical temperatures that remain constant across the Permian–Triassic boundary. The Early–Middle Triassic results, however, indicate remarkable differences between each area in paleo-LST variance through time (Fig. 4g).

Paleosols of likely Induan age from Graphite Peak and the Allan Hills display an overwhelming decrease in organic carbon content and a more limited variation in the types of soil horizons as compared to the late Permian paleosols in the same areas^11,16^. These properties are consistent with soil formation that lacks significant plant productivity. Here, a well-developed early Middle Triassic paleosol from the Allan Hills is studied in detail for paleo soil-forming processes and geochemistry (supplementary files 1, 4). The geochemical results of this paleosol are significant as this paleosol profile represents the stratigraphically lowest evidence of significant pedogenic alteration of the land surface following the Early Permian record of incipient soil formation. Therefore, this profile may record pedogenesis on these landscapes in the early stages of re-establishment of forested ecosystems in the Transantarctic Basin. Major element abundances of the Triassic paleosol display down-profile trends of Ca, Mn, Al, K, and Mg, with slightly variable Na abundance. The abundance of Mn is depleted in the B horizons but highly abundant in the parent material where Mn-nodules were observed in the field. The abundance of Ca is notably depleted in the B horizons of this paleosol relative to the parent material; whereas Al, K, and Mg are abundant in the B horizons and display a gradual decline towards the parent material. For application of paleoclimate proxies the CIA-K value maintains a > 5% difference between the parent material and overlying B horizons, indicating the likelihood that the geochemical effects of soil-forming processes are preserved in this paleosol.

## Discussion

The data presented here provides direct evidence of the response of plants to climate in the late Permian and early Middle Triassic through high-resolution analysis of paleoclimate data at discrete time intervals within the stratigraphic successions studied herein. For context, the late Permian ecosystems of Antarctica were low-diversity forests with arborescent taxa dominated by the glossopterids. Despite low generic diversity, however, isotopic data indicate varied functional diversity of glossopterids in the form of leaf habit^35^ and likely a greater species richness of glossopterids based on their reproductive organs^36^. These ecosystems were long-lived on Gondwana^9,10^, forming the predominant vegetative cover for the Permian after the end of the late Paleozoic ice age. Following the late Permian, evidence from the Sydney and Bowen basins indicate prolonged environmental disturbance resulting in the predominance of toxic algal blooms in freshwater settings^37^. The ecologic recovery from this disturbance in early Middle Triassic preserves a much higher diversity of arborescent and herbaceous vegetation^15^, with the re-emergence of pteridosperms in the Transantarctic Basin in the form of *Dicroidium* and associated corystosperm wood morphogenera^38^. The paleoclimate change accompanying these ecologic shifts is discussed here in two temporal scales and over a range of paleolatitudes. Long-term paleoclimate change is inferred from the Sydney, Bowen, and Transantarctic basins through analysis of major element concentrations in sedimentary rocks and paleosols^18,19^. The dendrochronology data herein provides “snapshots” on a centennial timescale for the organismal response to paleoclimate at a given time interval. Broad comparisons between low-latitude and high-latitude climate are addressed through comparison of eastern Pangea successions of sedimentary rocks and paleosols^28,39,40^ to the aforementioned strata.

What were the specific changes to paleoclimate? Changes in atmospheric circulation and an increase in humidity likely explain the long-term paleoclimate averages of sediment geochemistry data^19^. The paleosol geochemistry data (Fig. 5e) presented herein confirms that assessment, for long-term averaging of paleoclimate information. However, the dendrochronology data herein is presented at annual resolution, which has the potential to highlight specific climate change mechanisms. These results indicate a shift in internal climate oscillations from decadal to sub-decadal in the early late Permian to multidecadal oscillations in the latest Permian and early Middle Triassic. Without suitable comparison to annually resolved paleoclimate simulations, it is speculated that oscillatory phenomena, akin to the extant Arctic annular oscillation (AO) is a plausible atmospheric–surface ocean modern analogue that may explain some of the oscillatory behavior observed in the deep-time tree-ring chronologies. Given that AO, like our CWT results, is non-stationary and does not occur at a fixed periodicity and occurs at high latitudes. Furthermore, it is expected that because of declining hemispheric temperature gradients (Fig. 4f), the oscillatory climate behavior similar to AO may have weakened substantially by the latest Permian given the increase in multidecadal climate oscillations in tree-ring records at this time. The lengthening of the period of the internal oscillations of climate indicates that the impact of changes in rainfall or snow accumulation on this more expanded climate oscillatory framework negatively impacted tree growth of the glossopterids at these paleolatitudes (Fig. 5c).

Alternatively, the northward drift of Gondwana has been invoked to explain the observed changes in climate and flora through the late Permian–Triassic^41^. However, more recent paleogeographic reconstructions^42^ indicate that the study region likely moved northward by ~ 5° of latitude (85° S to 80° S paleolatitude). Therefore, the motion and position of Gondwana is not consistent to explain the observed climate changes or changes in internal climate oscillations. The climate changes observed herein, during the latest Permian are subtle, which is a similar result as reconstructed for the Sydney and Bowen basins^4,14^. The subtlety of these climate changes, here pinpointing a shift in internal climate oscillations rather than shift in climate regime, further indicate that perturbations to the Earth surface system (atmosphere, ocean, biosphere) are the most parsimonious mechanism to further understand these results.

The CWT analysis presented here uses the Morlet wavelet (Fig. 4a–f), which is useful for detecting oscillatory signals and their stationarity in a time series. However, because the Morlet wavelet uses real and imaginary numbers, the wavelet power includes information about amplitude and phase, hence resolution at fine-time scales is sacrificed for accuracy of the frequency domain^33^. By selecting a derivative of a gaussian wavelet (DOG, 2nd order derivative), the resolution of the time scale conforms to the resolution of the original data set, due to visualizing positive and negative oscillations as separate peaks, allowing for direct comparison to be made of wavelet coefficients to the RWI for a given year (Fig. 5a–d). Of the growth years that correlate to significant wavelet coefficients in the latest Permian chronologies, 60–62% correspond to years of suppressed growth, mostly around the 30 years periodicity. In contrast, only 40% of the growth years in the early Middle Triassic correspond to suppressed growth, also mostly around the 30 years periodicity. Thus, despite similar patterns of oscillatory paleoclimate in the latest Permian and early Middle Triassic, the plant communities responded in vastly different ways to this climate state, with the latest Permian glossopterid forests being indicative of a highly stressed ecosystem.

Longer-term averages of paleoclimate information are derived from the morphologic and geochemical analysis of paleosols and sedimentary rocks (Figs. 4g and 5e)^18,19,28,39,40^. The late Permian witnessed an increase in land surface temperatures (LSTs) by 10 °C or more^19^, values consistent between eastern Australia and Antarctica. However, the paleotropical North China craton sedimentary record shows a comparatively muted temperature increase of ~ 5 °C^28^ (Fig. 4g). A brief time interval across the Permian–Triassic boundary, however, was markedly devoid of LST variation in all areas. The Induan paleosol record from Antarctica indicates that during this timeframe of invariant LST, the soil-forming environment produced morphologically immature soil profiles with minimal organic carbon content. The greater range of temperature increase in the paleopolar regions of Gondwana may indicate that wildfires^43^ could have become a more important source of ecologic disturbance during the Lopingian. Evidence for paleofire on Gondwana is based on physical observations of inertinite coal macerals and/or charcoal fragments, or through geochemical identification of polycyclic aromatic hydrocarbons (PAHs)^43^. Inertinite abundances, however, were initially interpreted to reflect “freeze-drying” of humified biomass in the presumed cryic–gelic (0°–8 °C, < 0 °C mean annual soil temperature) temperature regime of Gondwana^44^. Refinement of paleoclimate interpretations for the Lopingian on Gondwana do not support a cryic–gelic temperature regime^4,18,19^, therefore fire is the most parsimonious interpretation of anomalous inertinite abundances in Gondwanan coals^43^. Inertinite abundances in a continuous succession of Lopingian–Induan strata in eastern Australia and the Lambert Graben, East Antarctica provide a higher-resolution record of potential fire activity during this time interval^45^. This record indicates a prevalence of burned plant tissues from above-ground biomass in well-drained paleoenvironments, in addition to minor amounts of burning of below-ground biomass in wetland paleoenvironments. Volcanism is invoked as a probable ignition mechanism for wildfires on the paleolandscapes of eastern Australia. If a similar record is anticipated for these Antarctic successions, then it is likely that the Shackleton Glacier Area (CTAM) would have had a similar ignition trigger for wildfires as eastern Australia, where both areas underwent a similar trajectory in climate change. However, fire, if interpreted as a single mechanism that contributed to the extinction of the glossopterids, is complicated by the relatively surficial burning of glossopterid biomass^45^, and by the trajectory of increasing humidity of these areas during the Lopingian and into the Induan (i.e., an increase in the annual balance of rainfall versus evapotranspiration). In addition, the early Middle Triassic of the SVL area preserves evidence for the persistent re-occurrence of wildfire affecting above-ground biomass in well-drained paleoenvironments^46^. However, this newly resolved record of paleo-wildfire from eastern Australia^45^ underscores the need for continued high-resolution stratigraphic and paleoecologic analysis of wildfire to better understand the role that fire may have played in the fate of the glossopterid biome.

Paleosol geochemistry from previous studies^16,18,47^, when applied to a paleoclimate proxy^31^, indicate that late Permian paleoclimate established a humidity gradient between SVL and CTAM (Fig. 5e). These results stand in stark contrast to the overwhelming evidence for aridification in the paleotropical latitudes during the Late Permian^28,38–40^, indicating that while the evidence for warming is consistent for terrestrial and marine strata, the effect on climate was likely related to zonal patterns in atmospheric/surface ocean circulation. The results of paleosol geochemistry for this study indicate that the early Middle Triassic paleosol from the Allan Hills formed under a humid and afforested biome, consistent with the production of clay and Fe-oxide minerals in the studied profile and lack of evidence of forested soil morphology in early Triassic paleosols in the Transantarctic Basin^11,47^. The “rainforest” designation for these paleosols indicates the possibility that closed canopy forests could have been maintained in these environments given the balance of rainfall relative to evapotranspiration. Fossil forest reconstructions from this region^17,48^ lend support to this interpretation based on the relatively high tree density per unit area and basal area of wood. However, the latest Permian and the early Middle Triassic paleosols of both study areas converge to the same humidity and floral province biome as the paleosol studied herein. These results are consistent with the interpretations of the dendrochronology time series, where distinct changes in oscillatory behavior are observed to coincide with prominent shifts in LST during the Late Permian, where the Middle Triassic displays an overall similar paleoclimate in the study region.

The Sydney and Bowen basins were adjacent to the study area during the late Permian–Middle Triassic. Recently, it has been hypothesized that terrestrial ecosystems underwent an ecologic collapse prior to the marine-defined EPE^4,49^, and that paleoclimate was likely the driver of ecosystem collapse and restructuring^14,19,37^. Based on this hypothesis it would be expected that contiguous ecosystems at higher paleolatitudes would be equally sensitive to the same climate forcing. This study, therefore, evaluates this hypothesis through the record of paleoclimate change in the plants that went extinct during the late Permian. The shift in internal climate oscillations from the late Permian to latest Permian fossil wood confirms a climate change event during the time interval preceding ecosystem collapse at the paleo high-latitudes. Coincident with the change in climate oscillation is an increase in rainfall relative to evapotranspiration, indicating these climate oscillations likely influenced the moisture balance in this region. Moreover, the correlation of this 30 years oscillation with years of suppressed growth in the Permian, in contrast with correlated enhanced growth in the Triassic, further supports the implication that these changes in climate oscillation and moisture balance in late Permian paleoclimate were deleterious to terrestrial ecosystems in the high-paleolatitudes on Gondwana.

## Conclusions

This study documents the history of tree-ring growth at paleopolar latitudes from the late Permian–early Middle Triassic in order to evaluate the hypothesis that paleoclimate change was a principal cause of the demise of glossopterid ecosystems. Dendrochronologic results are statistically robust and highlight a change in the period and stationarity of oscillatory climate effects on tree-ring growth in the study area. Geospatial comparisons of dendrochronologic results indicate a subtle gradient existed between the two study regions, with the gradient decreasing into the early Middle Triassic, consistent with long-term averages of paleoclimate derived from paleosol climate proxies. Latest Permian tree-ring chronologies are markedly similar to the early Middle Triassic chronologies, with a key difference being the correlation of a 30 years signal with years of reduced growth for Permian trees and a correlation of a 30 years signal with years of enhanced growth for Triassic trees. These results add support to the hypothesis that paleoclimate exerted significant stress to terrestrial ecosystems during the late Permian and that these stressors occurred in advance of the marine record of the end-Permian extinction.

## Methods

### Study sites

The fossil wood used in this study was collected from Permian and Triassic strata of the Transantarctic Basin in the CTAM and SVL regions of Antarctica. Each sample reflects fossil wood material collected directly from a sedimentary bed, preserved either in life position (in situ) or as an allochthonous fragment of woody debris. For each sample set, fossil wood was collected and processed for dendrochronology from the same sedimentary bed in order to minimize erroneous cross-matches of fossil wood from older/younger sedimentary deposits. Late Permian fossil wood was collected from the upper Buckley Formation at McIntyre Promontory (CTAM, S84°55.168′, E179°43.182′). Fossil wood stratigraphically close to the Permian–Triassic transition was collected from the lower Fremouw Formation at Shenk Peak and at Collinson Ridge (CTAM, S85°13.275′, W173°57.704′; S85°20.051′, W175°28.218′, respectively). Of these samples, only fossil wood at Collinson Ridge is preserved as in situ fragments. The remaining wood samples are preserved as horizontal to subhorizontal wood fragments in sandstone/siltstone strata. Fossil wood material was collected from specimens where > 20 tree-rings can be identified (~ 10 cm, or greater, diameter), a minimum number of rings for replicable cross-dating. Comparisons are made to previous dendrochronologic results from the Permian Weller Coal Measures (S76°42.577′, E159°42.826′) and Triassic Lashly Formation (S76°40.524′, E159°52.203′), Allan Hills (SVL^17^).

### Paleobotany

Tree rings identified in hand sample are cross-referenced to thin-sections of the transverse and radial planes of fossil wood. The taxa studied include glossopterid wood from upper Permian successions in CTAM and SVL, which are dominated by woody axes with affinity to the glossopterids, and are associated with megafloral remains of *Glossopteris* leaves, *Vertebraria* roots, and reproductive organs related to the glossopterids^50^. The Permian fossil wood studied herein preserves elements of wood anatomy consistent with wood morphogenera associated with the glossopterids, such as *Australoxylon*. The early Middle Triassic, however, contains a more diverse megafloral community of arborescent plants. Distinguishing wood morphogenera between conifers and corystosperms is challenging due to the conservative nature and few unique properties to distinguish these taxa^38^. However, while not conclusive, the early Middle Triassic wood studied herein was collected from sedimentary beds containing an abundance of *Dicroidium* leaf compressions and the woody axes display the prominent lobed property associated with corystosperm fossil wood.

### Dendrochronology

The techniques of dendrochronology (statistical cross-matching of tree ring widths, TRW) have been successfully applied to fossil wood material in deep time in order to generate robust annual chronologies of wood growth^17,51–53^. Here, these techniques are applied to Permian and Triassic fossil wood to study the paleoclimate history during the time of this wood growth. As climate is among the most important state factors for tree growth^27^, and that these extinct trees grew near the limit of their natural range, it is expected that paleoclimate information is encoded in the TRW variation of these samples. Thin sections of the transverse and radial planes were made from each sample to assess the preservational state of the fossil wood material; and relate the anatomically defined ring boundaries to their macroscopic expression in hand sample. TRWs were measured by hand with high-precision calipers under 10× magnification, with measurements of TRW at a precision of thousands of a millimeter. For each sample a minimum of two radial transects were measured to ensure replication of the TRW measurements. Replicate transects were cross-matched, and averaged if a successful cross-match was made. Several samples were measured independently by two to three analysts. Cross-matching was performed using PAST5™ software on: replicate TRW transects of a sample (intra-tree cross-matching); and between different samples (inter-tree cross-matching). Intra-tree and inter-tree cross-matching was assessed via statistical comparison of: (1) t-statistics (T_BP_^54^; T_HO_^55^); (2) the correlation coefficient; and (3) percent parallel covariation. Additional considerations on accepting or rejecting a cross-match include: (1) the number of overlapping rings; (2) the number of inter-tree cross-matched samples at unit chronology length (sample depth); and (3) anatomical constraints on internal cross-matching. Detrending of the raw TRW data to remove spurious growth-related trends in TRW was performed with a smoothing spline. The detrended master TRW chronology is converted to the Ring Width Index (RWI), which is an index relating the measured TRW to the expected TRW for a given ring number. RWIs have a value that is either < 1 (less growth than expected), = 1 (expected growth), or > 1 (more growth than expected). The potential that a segment of a chronology preserves the total expression of a climate signal in a TRW chronology is assessed statistically via the subsample signal strength (SSS) with dplR in R^56^ with a cutoff value of 0.5 arbitrarily chosen to represent robust inter-series correlations^57^.

### Continuous wavelet transform

Given that annually resolved meteorologic variation in deep time is not widely reported, and that the studied taxa are extinct with no viable modern counterpart for comparison, the periodicity of RWI variation via continuous wavelet analysis is used to infer the response of the studied fossil wood to oscillatory paleoclimate variation. However, each RWI chronology must have a Gaussian distribution in order to be used in a continuous wavelet transform (CWT). Probability plots (supplementary Fig. 3) of RWI chronologies are used to determine the type of statistical distribution the data has prior to CWT. A CWT decomposes a time series to provide information about specific frequencies, their variation through time, and significance level^33^. The Morlet wavelet was selected as an appropriate choice due to its: (1) simplicity; (2) widespread use; and (3) that it contains real and imaginary values for phase and amplitude comparisons^33^. Significance tests of the resulting wavelet power spectra were performed in R using the dplR^56^, and Wavelet Comp packages^58^. Significance tests evaluate wavelet power against the null hypothesis of a background spectrum of red noise, produced via an autoregressive process. Wavelet power that exceeds the red noise spectrum at the 5% significance level is interpreted to be a true feature of the data. Wavelet scalograms (Figs. 3a–d and 4b,d) were produced in Mathematica.

### Reproducibility

CWT results from two independently measured (by authors ELG and VC) TRW chronologies from the Triassic Lashly B member, Allan Hills (SVL) are compared via the technique of cross-wavelet transform to produce the cross-wavelet power spectrum and wavelet coherence^33^. This technique is applied here, via the Wavelet Comp package in R, to evaluate how reproducible TRW chronologies are in deep time as the fossil wood for both chronologies was sampled from closely spaced locations on two vertically associated bedding planes in the Lashly Formation in the Allan Hills. Thus, although minor differences are expected, the closely spaced nature of these samples in the sedimentary strata suggest a similar distribution of TRW signals should result, without the potential to provide meaningful cross-matches. The cross-wavelet power spectrum provides information about covariance at each unit time between two time series, with higher cross-wavelet power indicating a common power between the two time-series. The wavelet coherence provides information on the correlation of two time series per unit time, regardless of the power, providing information on how well-correlated two time series are. Coherence values approaching 0 indicate poor correlation, and coherence values approaching 1 indicate high correlation at the specified significance level. The phase of the covariance is reported for the cross-wavelet power spectrum and the wavelet coherence to inspect whether a correlation is positive or negative or whether one time series leads the other.

### Paleosol geochemistry

Paleosol morphology and major element geochemistry are used to provide an independent assessment of paleoclimate in the study region from the Permian and Triassic. Paleo-rainfall estimates are derived from the CIA-K proxy^32^, and paleohumidity and floral province inferences are derived from a separate proxy^31^. For this study major element geochemistry is reported from a well-developed gleyed ferritic Argillisol (iron- and clay-bearing paleosol with a prominent zone of reduced or oxidized material) profile that was described and sampled in the Lashly A member, Allan Hills (SVL)^17^. Paleosol morphology was described in the field at the cm-scale, and representative samples of the five identified subsurface horizons and parent material were collected. A bulk subsample from each horizon was ground, homogenized via mixing, fused with Li-metaborate, and dissolved in 6 N HCl. 20-fold diluted aliquots were analyzed by ICP-MS (Agilent 7700) at Gustavus Adolphus College (USA) for Al, Ca, Mg, Na, Mn, and K. Elemental abundances are converted from weight percent to moles for use in the paleoclimate proxies. Published paleosol morphology and geochemistry^16,18,47^ from the Permian and Triassic of CTAM and SVL are used to provide a complete and comparable paleosol-derived paleoclimate record in the study area.

## Supplementary Information

Supplementary Information 1.

Supplementary Information 2.

Supplementary Information 3.

Supplementary Information 4.

Supplementary Information 5.

## Data Availability

All data generated or analyzed during this study are included in this published article and its supplementary information files.
