# Supplementary material for: Paleoclimate-induced stress on polar forested ecosystems prior to the Permian–Triassic mass extinction
Source: Sci Rep. 2022 May 24;12:8702. doi: 10.1038/s41598-022-12842-w (PMC9130125; doi:10.1038/s41598-022-12842-w)
Supplement: Supplementary file 1 — Supplementary Information 1. [file 41598_2022_12842_MOESM1_ESM.pdf]

Paleoclimate-induced stress on polar forested ecosystems prior to the Permian–Triassic mass extinction

Erik L. Gulbranson[1*], Morgan M. Mellum[1], Valentina Corti[2], Aidan Dahlseid[1], Brian A. Atkinson[3], Patricia E. Ryberg[4], Gianluca Cornamusini[2]

[1]*Department of Geology, Gustavus Adolphus College, 800 W. College Ave, St. Peter, MN 56082 USA*
[2]*Dipartimento di Scienze Fisiche, della Terra e dell'Ambiente, Università di Siena, Italy*, I-53100
[3]*Department of Ecology and Evolutionary Biology, University of Kansas, 6012 Haworth Hall, Lawrence, KS 66045 USA*
[4]*Department of Physical and Natural Sciences, Park University, Parkville, MO 64152 USA*
[*]*corresponding author* Email: erikgulbranson@gustavus.edu

Supplementary Information and Data

1. Field descriptions and photographs

Shackleton Glacier Area, Central Transantarctic Mountains

McIntyre Promontory. McIntyre Promontory is a significant ridge of Upper Permian, Lower Triassic, and Lower Jurassic strata, with a ~30m dolerite sill emplaced in the middle of the slope. The Upper Permian Buckley Fm. is exposed as rock outcroppings from the base of McIntyre Promontory towards the ridgeline. The contact of the Upper Buckley Fm. and Lower Fremouw Fm. occurs roughly 10–30 meters below the ridgeline. The Lower Jurassic Prebble Fm. occurs as a small lens of strata preserved near the summit of McIntyre Promontory, recognized by heterolithic clasts of Buckley Fm. and Fremouw Fm. material in addition to mafic volcanic lithic fragments. An *in situ* fossil forest was discovered in the Upper Buckley Formation in the 2016–2017 austral summer season near one of the outcrop exposures below the sill. However, these fossil wood axes contain too few growth rings to warrant dendrochronologic analysis. The Upper Buckley Fm. outcroppings above the sill, however, contain numerous transported glossopterid wood axes (Supplemental Figure 1a–c), with sufficient numbers of growth rings for dendrochronologic analysis. The Upper Buckley Fm. at McIntyre Promontory is coal-bearing succession of volcanic lithic arenites and wackestones. Multiple pyroclastic flow deposits are interbedded in the lowermost units of the Upper Buckley Fm. (below the sill). These pyroclastic flow deposits contain a range of characteristic facies: basal surge deposit consisting of poorly sorted coarse-sand to pebble lithic fragments and welded lapilli; an overlying ripple-laminated to combined-flow laminated volcanic-lithic arenite; and an uppermost massive, polygonal-jointed air-fall tuff deposit. Samples were collected for future work on calibrating this stratigraphy to the geologic timescale via U-Pb LA-ICPMS/CA-IDTIMS analyses on zircon. Sandstones that form ridges in the slope are tabular and laterally continuous over much of the outcrop area and consist of vertically and laterally arranged macroform elements. Lenticular beds with erosive basal contacts are diffusely interbedded in these tabular outcrops. Sandstone-coal-pyroclastic flow successions are recessively weathered into the slope, where the sandstone constituents are laterally discontinuous over 10's of meters, grading into organic-rich shale. *In situ* stumps, *Glossopteris* leaf impressions, and *Vertebraria* axes are preserved in these laterally

discontinuous sandstones and organic-rich shales. Aside from the deposition of volcanic ash, the arrangement of sedimentary strata in the Upper Buckley Fm. at McIntyre Promontory indicates two distinct styles of fluvial deposition: 1) a prominent low-sinuosity and highly braided system that produced the prominent ridges of multistoried sandstone; and 2) a smaller-scale higher-sinuosity system, with less braided character and forming single-storied channels. The two facies likely represent a mosaic of landforms operating concurrently, indicating that the overbank environment contained wetlands and small sinuous stream systems adjacent to much larger low-sinuosity fluvial systems. The uppermost sections of the Buckley Fm. (above the sill) are dominated by the ridge-forming multistoried sandstones, with minor occurrences of poorly developed Protosol material separating packages of sandstone macroforms and lenticular sandstones. Abundant transported fossil wood, including very large fragments of stumps with attached root flare (Supplemental Figure 1b) occur near the basal contacts of individual macroforms. Samples of these transported wood fragments are used to construct the tree ring width chronology for McIntyre Promontory. The overlying Lower Fremouw Fm. is recognized as the first occurrence of prominent redoximorphic colors in paleosols and a change in sandstone composition, with a greater proportion of quartz clasts than the underlying Buckley Fm., and a dearth of volcaniclastic material.

Collinson Ridge. The Collinson Ridge field location is a small ridge extending away from a prominent cirque in the Halfmoon Bluff area of the Shackleton Glacier region (Supplemental Figure 1d). A dolerite sill demarcates the outcrop edge of the shallow sloping terrain of sedimentary strata that culminates in a small ridge (Collinson Ridge). At the upper contact of the sill a thin outcrop of the Upper Permian Buckley Fm. is exposed. Numerous concentrically laminated ferruginous cements ~2m in diameter are exposed at the land surface in the volcanic lithic arenites at this contact, indicating a high probability of extensive hydrothermal alteration during emplacement of the sill. The Upper Buckley Fm. at Collinson Ridge contains an assemblage of laterally continuous sandstone at the base, followed by a succession of laterally discontinuous sandstone, coal, and pyroclastic flow material. The latter elements contain abundant woody debris, *Glossopteris* impressions and *Vertebraria* axes, and one forest with six *in situ* stumps. The overlying, quartzose sandstones of the Fremouw Fm. contain abundant Upper Permian megafloral assemblages of *Glossopteris* impressions, numerous *Verterbraria*-rooted paleosols and myriad sandstone beds containing transported fossil wood. Thus, based on megafloral preservation at Collinson Ridge it is recognized that the lithostratigraphic contact between the Buckley and Fremouw fms. is likely diachronous across the Transantarctic Basin. The Lower Fremouw Fm. sandstones form laterally continuous units at >10 m scale, consisting of multiple laterally adjacent and vertically stacked macroforms. Fossil wood material is preserved in all macroform elements, but the extent of in situ wood fragments is best evidenced by a single bedding exposure leading towards the nose of Collinson Ridge (Supplemental Figure 1d). These sedimentary bedforms and morphology are consistent with a low-sinuosity system with a highly braided character. Two additional in situ fossil forests are known from the Lower Fremouw Fm. at Collinson Ridge. The most extensive of which is highlighted in Supplemental Figure 1d, and contains at least 37 *in situ* stumps with many instances of transported wood. The *in situ* stumps are found near the upper surfaces of macroform elements, indicating the establishment of in-channel forests. The overbank facies includes *in situ* glossopterid wood into permineralized peat (Upper Buckley Fm.) and *in situ* wood in organic-rich shale. Thus, the widespread preservation of glossopterids across different sedimentary facies indicates that the

paleo land surface was nearly completed afforested, but with distinct niches of wetland glossopterids growing on peat (Upper Buckley Fm.); overbank glossopterid ecosystems forming on the floodplain environment; and in-channel islands with small glossopterid forests. The lack of peat in the Lower Fremouw Fm., despite the continued preservation of glossopterid organs, highlights a topic that deserves more intense scrutiny regarding the paleoecology of these plants in the lead up to the end Permian extinction. The largest of these fossil forests (n=37) was sampled for dendrochronologic analysis and stable isotope analysis. This forest occurs ~12 m below the first occurrence of *Thrinaxodon liorhinus* at Collinson Ridge.

Shenk Peak. An isolated outcrop of Lower Triassic strata is found in the col between Shenk Peak and an unknown peak near Mt. Kenyon. The Lower Fremouw Fm. is sandstone-rich, with interbeds and intraclasts of heavily bioturbated silty mudstone. Overall, this succession is highly fossiliferous, containing abundant transported fossil and *Vertebraria*-rooted paleosols in the lower ~20 m of the section, in addition to numerous vertebrate fossils. The sandstones are laterally continuous >10 m, tabular, and are composed of multiple vertically stacked macroforms. Small lenticular channels occur within the sandstone units. Highly root-turbated and bioturbated weakly developed Protosols separate multistoried sandstone units in this outcrop area, however these Protosols are relatively thin and are laterally discontinuous along strike, where erosional basal contacts of the overlying sandstone units merges with the underlying sandstone units. Intense root-turbation of these Protosols is unique to Shenk Peak (Supplemental Figs. 1g–h), however, the possibility that this is a taphonomic bias based on the composition of the paleosol material should not be discounted. Nevertheless, the root preservation in these paleosols permits a detailed analysis of root morphologies as a function of depth. Thin lenticular sandstone channels occur throughout the succession, in the finer-grained units and within the vertically-stacked macroform complexes of the main sandstone outcrops. These observations are consistent with a low-sinuosity fluvial system with high-braiding character. Given the close proximity to Collinson Ridge and nearly identical paleoenvironments, the plant megafossil section of Shenk Peak is likely at a similar stratigraphic position to the *in situ* forests at Collinson Ridge.

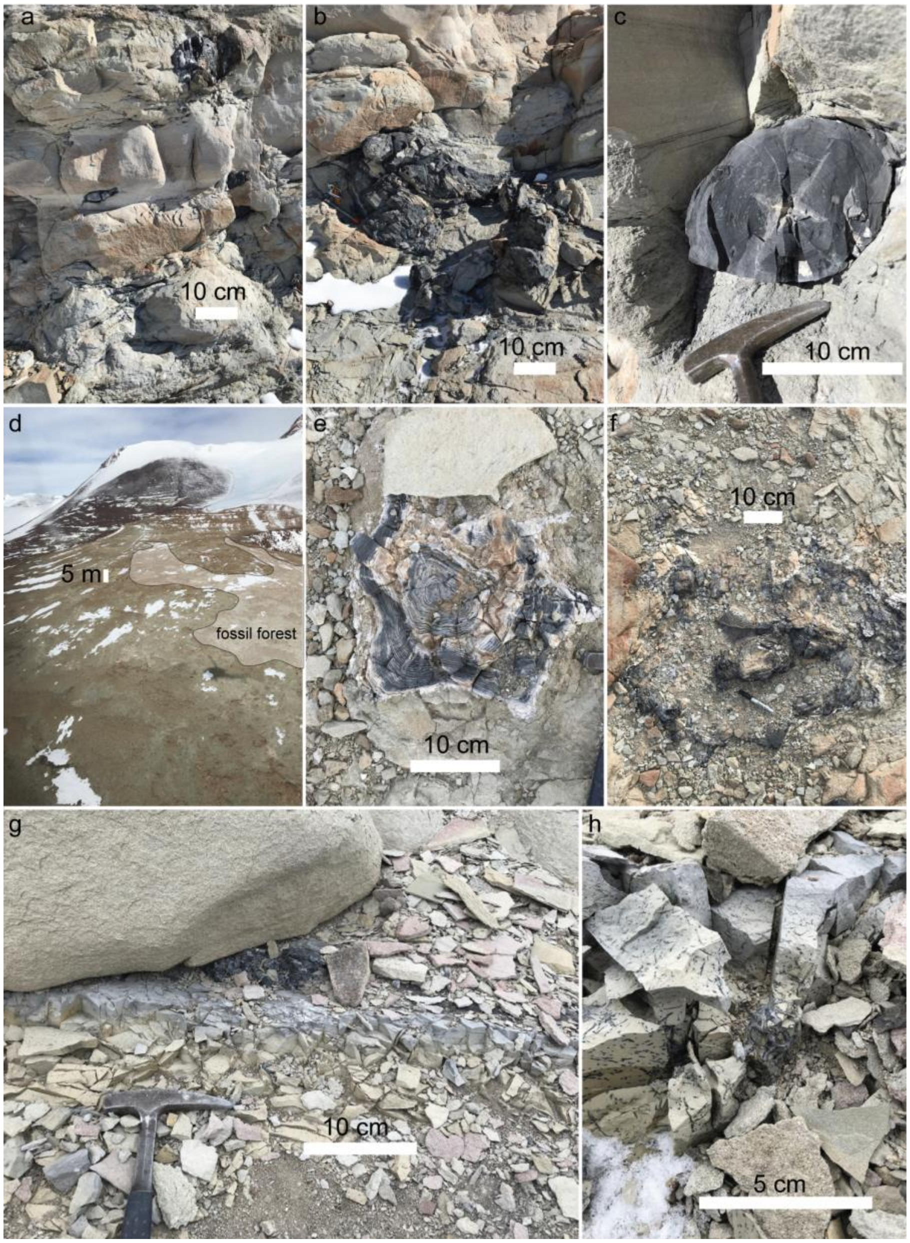

Supplemental Figure 1: Field images from the CTAM study area. Images a–c are of the Upper Buckley Fm., McIntyre Promontory. a) transported glossopterid wood axes, deposited near the base of 3rd order contacts of macroform elements within fluvial volcanic-lithic arenites and wackestones. b) transported glossopterid stump with root flare attached. c) transported glossopterid wood axes with detail of growth rings. Images d–f are of the Lower Fremouw Fm., Collinson Ridge. d) overview of the Collinson Ridge location, the broad plain in the foreground contains the *in situ* fossil forest studied herein (shaded). The broad plain connects to a small ridge, which contains the transition from Upper Permian strata to Lower Triassic strata based on megafossil preservation, inconsistent with the lithostratigraphic contact at this location. e) *in situ* glossopterid stem with growth ring detail. f) a large in situ glossopterid stem. Images g–h are of the Lower Fremouw Fm., Shenk Peak. g) transported fossil wood (dark region below sandstone boulder) on top of a *Vertebraria*-rooted paleosol. h) detail of the *Vertebraria* axes showing the classic lacunae infilled with coarse crystalline quartz. The smaller linear traces in the paleosol are rootlets that are densely dispersed in the upper 5 cm of the paleosol, the root diameter and density is used to demarcate soil horizons for this paleosol profile.

The Allan Hills, South Victoria Land

The Allan Hills. The systematic sedimentology of the study area in the Allan Hills is described in Gulbranson et al. (2020), including the occurrence of the woody debris and *in situ* stumps used for dendrochronologic analysis in this study. A summary of the key descriptions is provided here and in Supplementary Figure 2. Upper Permian strata in the Allan Hills include the Weller Coal Measures and likely a portion of the Feather Conglomerate. Lower Triassic strata are represented by a portion of the Feather Conglomerate and the Lashly Fm. (Supplemental Figs. 2a–b). The Weller Coal Measures is a sandstone-rich coal-bearing succession. Sandstone compositions are generally as arkosic arenites to wackestones, with variable amounts of organic carbon-rich laminations. The in-channel facies have an aspect ratio consistent with a ribbon sand morphology with a drastic change in grain size between the in-channel facies and overbank facies. These features are consistent with a low-sinuosity and braided river morphology. The ubiquitous presence of in-channel macroforms that host *in situ* fossil forests (Supplemental Fig. 2c) and evidence for wetland environments in the overbank setting (coal and permineralized peat) indicate an anastomosing fluvial geomorphology. Paleosols in the Weller Coal Measures include poorly developed Protosols (Supplemental Fig. 2d), which range from the floodplain environment to the afforested in-channel macroforms; and organic paleosols represented by the coal and permineralized peat. The Feather Conglomerate is a sand-rich succession of coarse-grained arkosic arenites with minor amounts of wackestone. Overbank facies and megafloral preservation are notably limited to absent, respectively, in the Feather Conglomerate. Paleocurrent data and in-channel morphology of macroforms indicates that the Feather Conglomerate represents deposition by low-sinuosity fluvial systems. Paleosols in the Feather Conglomerate are limited to poorly developed Protosols. The Lashly Fm. is divided into four members: A, B, C, and D. The A and D members are considered here. The Lashly A member consists of lithic arenites, wackestones; and silty mudstones. Relatively sandstone-poor in comparison to the underlying Feather Conglomerate, the sandstone units of the Lashly A Fm. are continuous at a scale <20 m and are consistent with a high-sinuosity fluvial system with little braided character. Isolated fragments of *in situ* fossil wood are found in the Lashly A Fm. sandstones. Paleosol of the Lashly A Fm. demonstrate a remarkable increase in soil development, relative to underlying stratigraphic units and a continuity of soil-forming processes (e.g., clay illuviation, weathering, iron-translocation) on the paleolandsurface. A gleied ferritic Argillisol was discovered in the Lashly A Fm. during the 2014–2015 austral summer season and the geochemistry of this paleosol is reported here as part of the long-term paleoclimate analysis. The Lashly B Fm. is relatively sandstone-rich, with tabular sandsheet morphologies continuous over the outcrop area. Interbeds of fine-grained overbank material, including organic-rich and fossiliferous shales occur as tabular beds or as tabular beds within a lenticular bounding surface, interpreted to be an abandoned channel. The Lashly B Fm. is interpreted as a lower sinuosity fluvial system with a high degree of braiding. Moreover, the overbank environment contained isolated higher-sinuosity fluvial systems, and the interpreted abandoned channel features. Abundant transported fossil wood occurs in the tabular sandsheet beds of the Lashly B Fm., in particular on a broad bedding plane exposed near an area referred to as Feather Bay, on the Allan Hills (Supplemental Figs. 2a–b). The transported fossil wood from the Lashly B Fm. was collected for dendrochronologic analysis reported in this study.

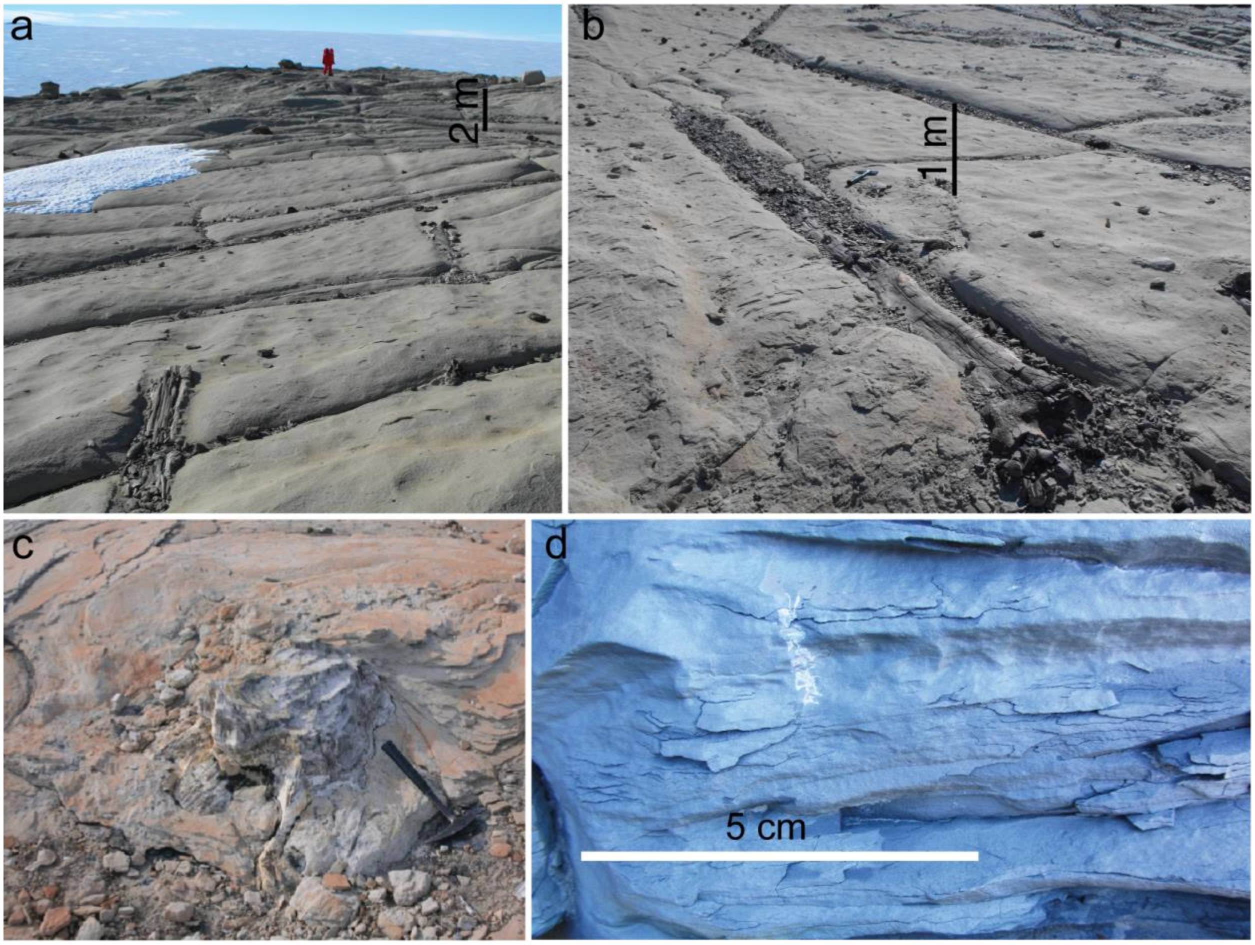

Supplemental Figure 2. Field images of the SVL study area. a–b) Transported fossil wood, longitudinal features exposed on the sandstone surface, Lashly B Fm., Feather Bay, Allan Hills. C) *in situ* glossopterid fossil wood on the upper surface of an in-channel macroform element, Weller Coal Measures, Trudge Valley, Allan Hills. d) *Vertebraria*, subvertically trending white object above the scale bar, on an incipient exposure surface near the margin of a channel, Weller Coal Measures, Trudge Valley, Allan Hills.

2. Cross-matches

Cross-matching of fossil wood is similar to the method of cross-dating applied to modern and subfossil wood. However, the key exception is that a cross-match will not confer a calendar year to a specific tree ring as the cross-match chronology is disconnected from a continuous tree ring chronology that extends to the modern age. Therefore, the time dimension of a cross-matched time series is relative; and it is vital to rely on stratigraphic principles to differentiate relative stratigraphic ages of fossil wood. Such that fossil wood from two different bedding planes is not cross-matched without additional geologic evidence to suggest the wood may have grown contemporaneously. The cross-matching procedure relies on accurate and reproducible tree ring width data as the fundamental data. Growth rings in 2 or more transects are measured for a given specimen, and the transects are cross-matched against each other, relying on anatomical criteria to assess the quality of a cross-match (e.g., pith, if present, must be aligned; bark-edge, if present, must be aligned). These "internal" cross-matches are expected to have exceptional statistics, but if the quality is poor this requires inspection of the problematic areas

and re-measuring some or all of the growth rings of a transect. Ring widths of high quality "internal" cross-matches are averaged at the specific position of the cross-match to produce an average ring width record of a sample. The average ring widths of two or more samples are subjected to the cross-match procedure, but in this process there is no *a priori* knowledge for the relative age of the samples. To carry out these cross-matches we conduct hypothesis testing, holding one ring width record (sample A) constant as a reference chronology and cross-matching a second ring width record (sample B) against it. The statistics of these cross-matches are equal to or worse than the "internal" cross-matches, reflecting tree-to-tree differences in growth conditions due to variations of one or more of the state factors for tree growth. If statistics and the number of overlapping rings of a cross-match between samples A and B is acceptable, this establishes the first hypothesis of the cross-match exercise: that sample B was growing at the specific relative time range determined by the cross-match over the growth history of sample A. a third sample (sample C) should be used to test the hypothesis in order to provide the most robust tree ring width chronology, and ideally the number of overlapping rings and overlapping cross-matched tree ring records should be maximized. This hypothesis testing procedure continues for all measured samples, resulting in one master chronology with significant cross-match statistics for all chronologies over a given time interval. PAST 5 software was used to perform these cross-matches due to its excellent visualization interface and rapid acquisition of key cross-match statistics. Raw ring width data and the cross-matched positions in relative time are provided in Supplementary File 2.

The master chronology is converted to a Ring Width Index (RWI) chronology by detrending growth related trends from individual tree ring chronologies. Growth related trends in tree growth can be easily envisioned by considering a hypothetical stem with an ideal conical shape. Where for each successive year of a growth a new cone is developed over the preceding one. For age-equivalent growth conditions over the lifespan of the tree, assume that the amount of biomass produced remains the same. Under this condition, the incremental difference in volume must change from one year of growth to the next. Thus, the incremental change in the diameter of the cones decreases over time, producing an age-related trend in ring widths if we examine a plane cut on the transverse plane of these nested cones. In reality, none of these ideal assumptions are met, however, the age-related growth trends still remain due to the outward growth of new woody tissue on previously laid down wood. The detrending procedure is a form of linear regression, often where polynomial functions best approximate tree ring growth over time. Thus, detrending with a spline is often encountered in dendrochronology, however, other regression functions may be used depending on the data type and/or length of the chronology. The detrending equation provides a prediction of the width of tree rings at a specific year for a given chronology, and is unique to that chronology. The measured ring widths provide the comparison to these predicted values, and the ratio of the measured ring widths over the predicted ring widths is the RWI. RWI values produced for individual chronologies are averaged at the specific positions in relative time based on the cross-match results, producing the master RWI chronology. The program dplR provides a convenient way to automate this procedure using cross-matched raw ring width data kept in an excel spreadsheet. The master RWI chronologies are provided in Supplementary File 2, and the dplR code (using the Collinson Ridge data file) is provided in Supplementary File 3 as an example for detrending and indexing raw ring width data.

The Subsample Signal Strength (SSS) metric is a comparison of correlation coefficients of segments of a chronology over the finite time range of the chronology, with a maximum value

of 1. An arbitrary cutoff value of 0.5 or greater for SSS has been widely used to determine chronology lengths that are robust and suitable for paleoclimate analysis. However, the determination and application of cutoff values for SSS and the expressed population signal (similar to SSS, but relative to a master chronology of infinite length) has been criticized as underestimating chronology lengths that are possibly valid for paleoclimate analysis (Buras, 2017). For this study we report the length of a chronology after which the SSS cutoff was exceeded. However, we do not factor these chronology lengths into our analysis of the continuous wavelet transform results, as this type of climate analysis differs from the more widely used method of correlating ring width data to specific climate metrics in addition to the aforementioned criticism of over-use of SSS.

Cross-match statistics. The dendrochronologic results of this study provide geospatial information for Late Permian tree growth at paleo polar latitudes, as well as temporal comparisons of paleo polar tree growth from the Late Permian to the early Middle Triassic. Late Permian chronologies across the study area display variable inter-tree correlations (supplementary Table 1), with *in situ* forests showing the lowest mean interseries correlation. One possible explanation for variable correlations is that glossopterid wood morphology is highly conservative[9] and may be affiliated with different glossopterid leaves and reproductive structures[27], which would potentially lessen the common growth signal expressed in the TRW records. Based on high-resolution carbon isotope analysis of fossil tree rings, glossopterids were either evergreen or deciduous trees, where the distribution of these leaf habits varied along paleolandscapes[28]. Functional diversity of this type could minimize the common expression of climate signals in TRW records, and perhaps explain the lower inter-series correlations, especially from *in situ* forests. For these reasons, dendrochronology of transported fossil wood may be preferable for future work, to limit paleoecologic artefacts, like functional diversity, in favor of maximizing inter-tree correlations and chronology length. RWI distributions of Late Permian chronologies display distinct spatial differences between the SVL and CTAM regions (Figs. 2a–d), which indicates dissimilar seasonal temperatures and rainfall amounts across the Transantarctic Basin.

Triassic chronologies display a wide range of inter-tree correlations (supplementary Table 2), which could relate to different corystosperm plants present in these chronologies with similar wood affinities, as possibly congeneric relationships between three different corystosperm wood morphogenera have been previously determined[29]. The distribution of RWI values of Triassic chronologies is nearly identical to the latest Permian chronologies, despite the chronologies coming from different regions of the study area (Figs. 2e–f). This similarity suggests possibly similar growing conditions during the early Middle Triassic as for the latest Permian.

Table 1: Statistics of cross-matches for Permian fossil wood, Antarctica

Location	Formation (age)	Number of samples	Cross-matched wood samples	Chronology length (yrs)	Average T_{BP}[1]	Average T_{HO}[2]	Mean Inter-series Correlation[3]	Average PPC[4]	Average overlap
Allan Hills	Weller Coal Measures	11	7	86	2.63	3.14	0.16 (-0.03–0.37	70.5%	24

McIntyre Promontory	Upper Buckley Fm. (Late Permian)	11	8	42	2.1	1.9	0.5 (0.15–0.78)	72.3%	20
Shenk Peak	Lower Fremouw Fm. (Late Permian)	6	5	113	2.3	2.4	0.62 (0.44–0.77)	73.4%	13
Collinson Ridge	Lower Fremouw Fm. (Late Permian)	7	7	176	1.7	1.7	0.26 (0.15–0.83)	68.6%	26

[1] T-statistic after Bailie and Pilcher, 1973
[2] T-statistic after Hollsetin, 1980
[3] Range of correlation coefficients are expressed in parentheses
[4] Percent parallel covariation

Table 2: Statistics of cross-matches for Triassic fossil wood, Antarctica

Location	Formation (age)	Number of samples	Cross-matched wood samples	Chronology length (yrs)	Average T_{BP}[1]	Average T_{HO}[2]	Mean Inter-series Correlation[3]	Average PPC[4]	Average overlap
Allan Hills	Lashly B Fm. (early Middle Triassic)	11	11	237	1.70	1.66	0.26 (-0.34–0.83)	68.6%	26
Allan Hills	Lashly B Fm. (early Middle Triassic)	7	7	238	3.38	3.11	0.39 (-0.13–0.84)	76.3	32

[1] T-statistic after Bailie and Pilcher, 1973
[2] T-statistic after Hollsetin, 1980
[3] Range of correlation coefficients are expressed in parentheses
[4] Percent parallel covariation

3. Distribution of RWI values

The distribution of RWI values for each chronology was assessed to: 1) gauge the suitability for continuous wavelet transform; and 2) as an additional metric to compare the RWI data sets across time and space. The RWI distributions for each chronology demonstrate a gaussian distribution, indicating that each chronology can be used for a continuous wavelet transform. However, subtle differences exist in the distributions between Permian chronologies from South Victoria Land (SVL) and the Shackleton Glacier area (CTAM); and between both Permian sample locations and all of the latest Permian–Triassic chronologies (Supplemental Fig. 3).

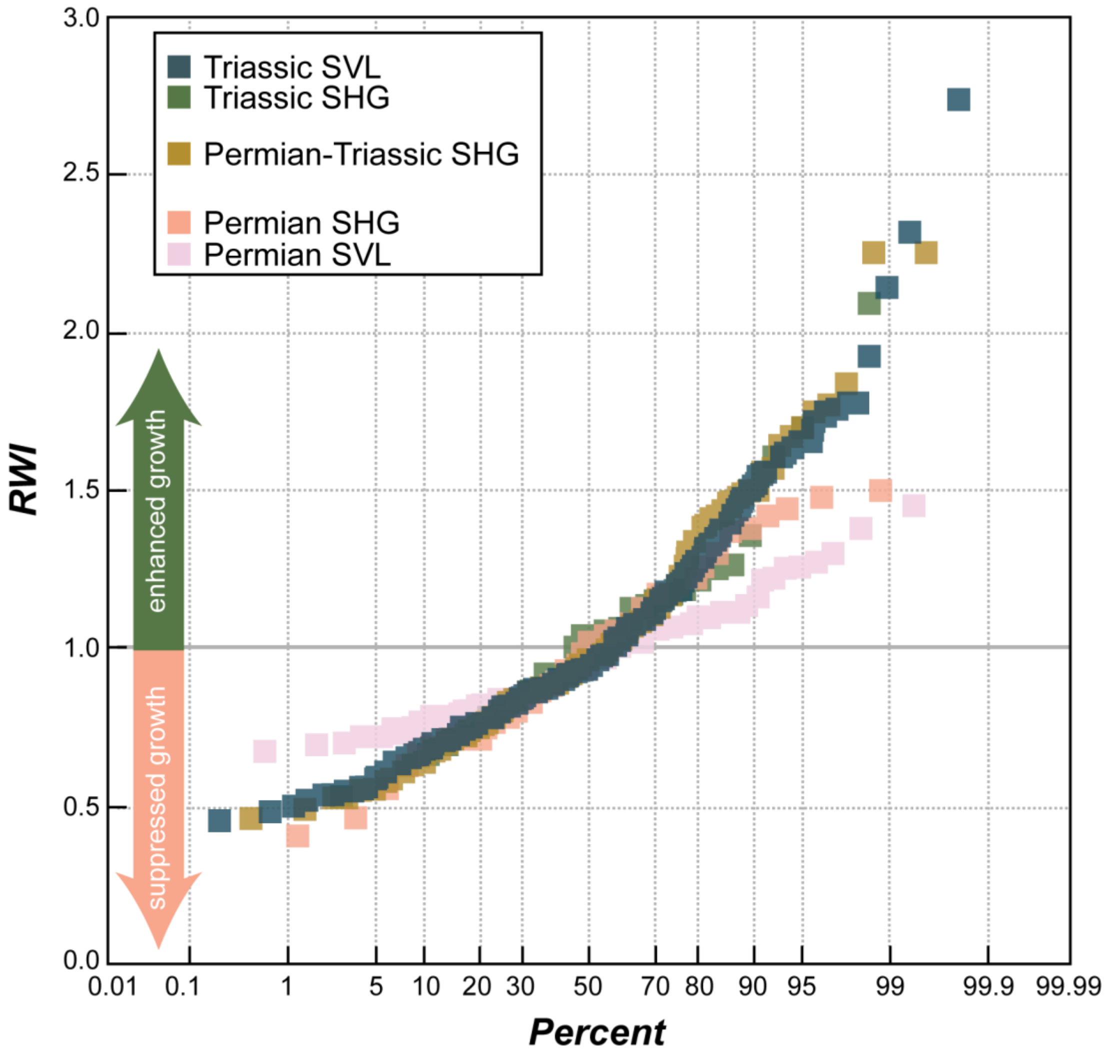

Supplemental Figure 3. Distribution of RWI values among the tree ring width chronologies. The interpretation of enhanced growth versus suppressed growth is illustrated by the arrow symbols about the RWI value of 1. The ages and locations of each tree ring width chronology is illustrated by the color of each symbol.

4. Wavelet Analysis

By inspection, RWI results exhibit nuanced periodicity of values greater than or less than 1 (Supplemental Figure 4). Moreover, this periodicity is complex, not being easily determined by graphical analysis or fitting a plane wave function to the data. As we hypothesize that RWI information predominantly includes a climate signal, it is of vital importance to extract these periodicities from RWI data to understand the oscillatory behavior of paleoclimate in these time series. Continuous wavelet analysis (CWT) is a robust approach to achieve this goal. A CWT analysis provides information for: 1) the range of frequencies in a given data set; and 2) how those frequencies change over the time recorded by the time series.

Stationary waves and non-stationary waves. One of the most basic questions a researcher can ask of a time series of data is whether there is a stationary signal or non-stationary signal. To visualize the difference of these two-end members it is convenient to imagine a sine or cosine wave with constant amplitude and wavelength. These sine or cosine waves are examples of stationary waves, in which case if we extract a frequency from these functions and plot that in

a frequency vs. time plot we would see a constant frequency over the length of time. Non-stationary waves, therefore, have episodic or periodic fluctuations in amplitude and wavelength. More than one frequency could be extracted from non-stationary waves, where those frequencies would plot over finite ranges of time on a frequency vs. time plot. If we imagine complex data where there may be both stationary and non-stationary waves superimposed on each other, then it becomes difficult to accurately deconstruct the data into its constituent signals by visual inspection alone.

Decomposition of a non-stationary wave into specific scales. To further visualize the power of a CWT for the analysis of time series we will use the Collinson Ridge RWI time series in the subsequent examples. The Collinson Ridge RWI is shown in Supplemental Figure 4.

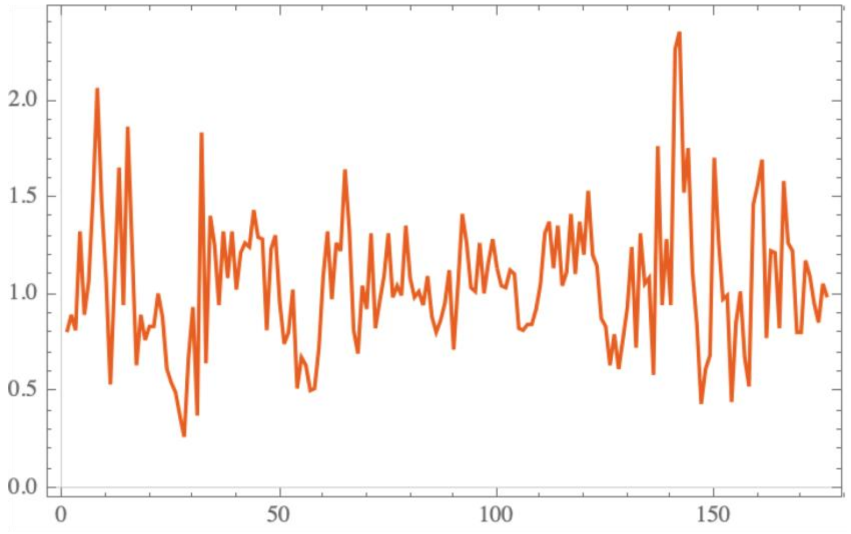

Supplemental Figure 4. RWI chronology for Collinson Ridge. The ordinate axis displays the RWI values on a scale ranging from 0 to 3. The abscissa displays the chronology length in units of years. The orange line is the average RWI for all of the cross-matched samples.

We apply a decomposition algorithm to this dataset using dplR (Bunn, 2010) (Supplemental Fig. 5). The decomposition arranges the data into the Fourier periods on the ordinate axis, which are equivalent to scales. Each scale is separated by 1 octave from the next. The intensity of the signal at each scale is plotted over the range of time for this time series. We can now visualize how the time series is constructed from the superimposition of these signals at different scales at specific time intervals in the time series. But, how can we distinguish true paleobiologic signals in this data from random noise? A CWT analysis has additional benefits not included in this wave decomposition: 1) by correlation of signal against the specific wavelet being used; and 2) evaluating the statistical significance of these signals against the null hypothesis of a red-noise spectrum.

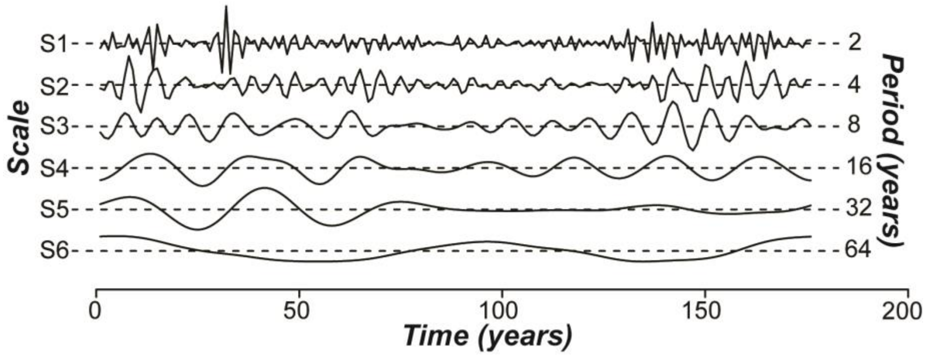

Supplemental Figure 5. Decomposition of Collinson Ridge RWI chronology into individual scales. The scale and associated Fourier period are shown on both ordinate axes. The chronology length is shown on the abscissa. The intensity has been normalized for all scales to more easily visualize all of the scales on the same image.

The continuous wavelet transform ($W_{x(u,s)}$) uses a small wave of specific frequency to map over all of the time series information at multiple scales (i.e., Fourier periods). The interaction of this wavelet with data preserves the energy of the original time series, and the result of the transform is the correlation of the wavelet to the data (wavelet coefficient, 0–1) and the wavelet power (square of the wavelet coefficient). The equation to scale a wavelet over multiple scales and at position, u, is as follows:

$$\psi_{u,s}\,(t) = \frac{1}{\sqrt{s}}\psi\left(\frac{t-u}{s}\right)$$

where, ψ is the wavelet, s is the scale, t is time, and u is the position of the wavelet. There are several options of wavelets to choose from. The Morlet wavelet (ψ_M, Supplemental Figure 6) is one of the most common forms to use for time series analysis, where the equation of the Morlet wavelet is:

$$\psi_M = \frac{1}{\sqrt[4]{\pi}} e^{iw_0 t} e^{-\frac{t^2}{2}}$$

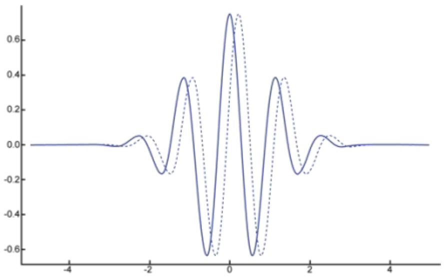

Supplemental Figure 6: The Morlet wavelet. Real numbers shown in solid line, imaginary numbers shown in the dashed line.

the central frequency, w_0, of the Morlet wavelet is selected to be 6. As the Morlet wavelet equation indicates, this wavelet has numbers mapped onto real numbers and imaginary numbers. Below, the equation for the CWT is shown. This equation can be read on the RHS as the time series (x, in green) evaluated by the wavelet ($\psi_{u,s}\,(t)$, in blue).

$$W_{x(u,s)} = \int_{-\infty}^{\infty} x(t) \; \frac{1}{\sqrt{s}}\psi\left(\frac{t-u}{s}\right) dt$$

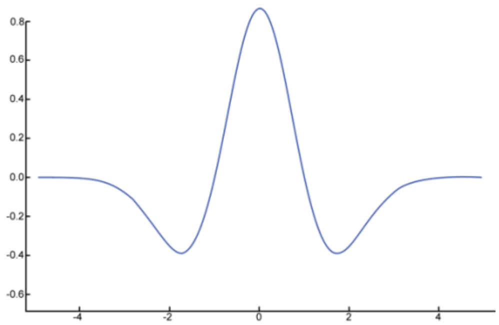

Supplemental Figure 7: The derivative of a Gaussian wavelet.

The Morlet wavelet is just one of several wavelets a user can select for CWT analysis. Where the Morlet wavelet is viewed as a powerful instrument to detect signals in the frequency-time domain. In this specific time series, however, we wish to understand if a given signal correlates to the productivity of trees in these fossil forests (e.g., RWI values >1 or <1). The Morlet wavelet is thus too broad to inform us of such precise (i.e., annual) correlations. Thus, we use the derivative of a Gaussian (DOG, Supplemental Figure 7) wavelet to analyze these tree ring time series in more detail. The DOG wavelet parses signals by their respective sign, which provides a finer-resolution on the time scale of the frequency-time domain. We use the conventional order of 2 (i.e., m=2) for the DOG wavelet in this analysis, as shown below.

$$\psi_{DOG} = \frac{(-1)^{m+1}}{\sqrt{\Gamma\left(m+\frac{1}{2}\right)}}\frac{d^m}{dt^m}\left(e^{-\frac{t^2}{2}}\right)$$

A key property of the continuous wavelet transform is the admissibility condition, which is that the inverse of the wavelet transform must completely reconstruct the time series from the wavelet transform. Supplemental Figure 8 shows an example, using the Collinson Ridge RWI, of how the choice of CWT scale affects the admissibility condition. On the left column (A–F) the CWT is shown at increasing ranges of scale from four octaves (Supplemental Fig. 8A) to nine octaves (Supplemental Fig. 8F). On the right column (G–L) of Supplemental Figure 6 the corresponding inverse wavelet transform (“iCWT”, blue) and original RWI data (“data”, orange) are shown. By increasing the scale number for the CWT relative to the chronology length we preserve more of the information of the original data set. However, the specific number of scales selected is based on the size of the dataset, with datasets varying by one order of Log_2 having 1 more or fewer scales, respectively.

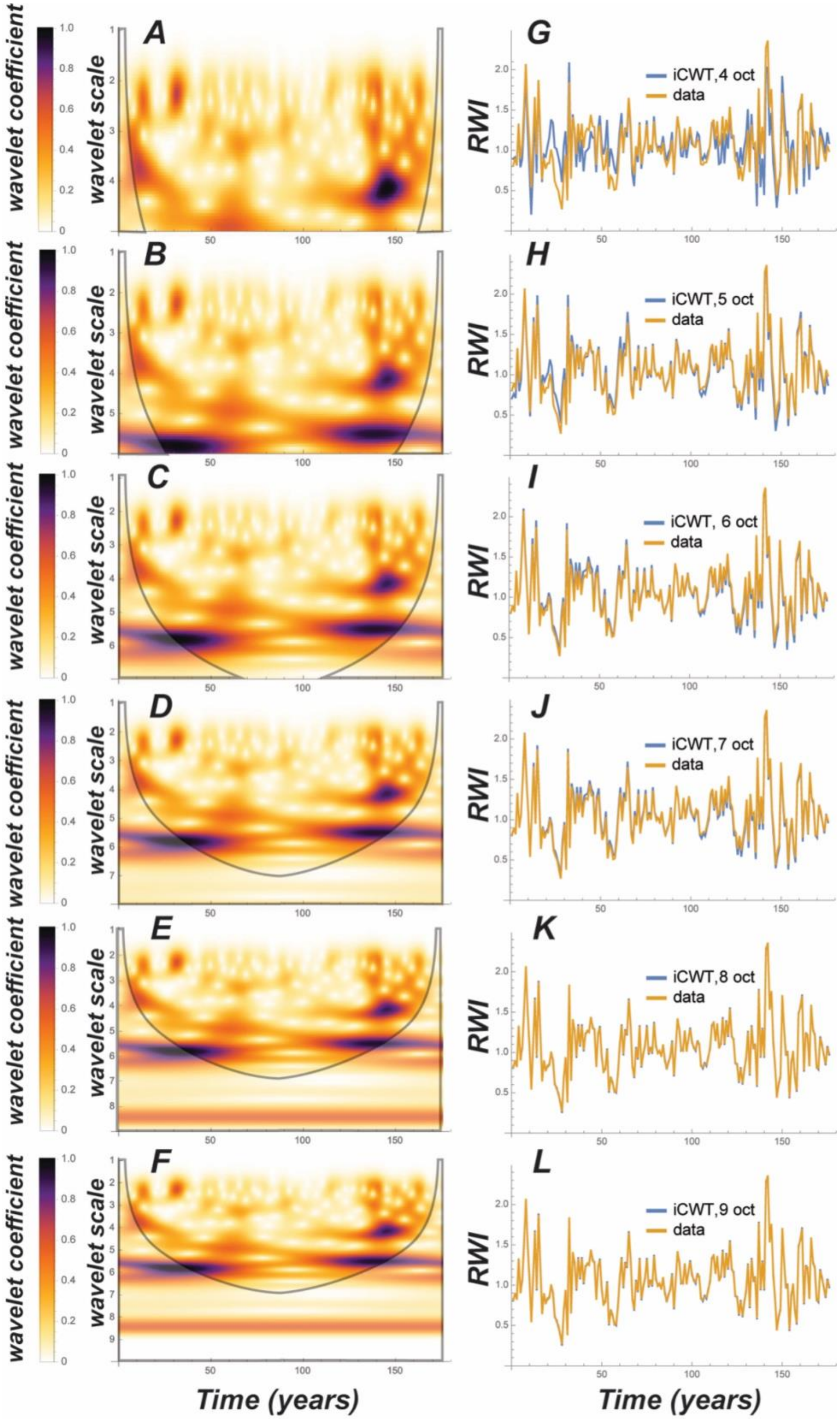

Supplemental Figure 8. Comparison of CWT and the fidelity of the inverse wavelet transform as a function of scale.

CWT results indicate that Late Permian chronologies in CTAM and SVL display subdecadal oscillations in climate, but with a distinct ~15 yr periodicity in the SVL chronology (Figs. 3a–b). The existence of the 15 yr period in the SVL chronology likely explains the subtle difference in RWI distributions observed in these results, corroborating a geospatial gradient in climate effects on plant growth over the study area. The latest Permian chronologies, however, are remarkably different with ~30 yr periodicity, and a lack of the 15 yr periodicity. High resolution analysis of the phase of these oscillations indicates that RWI values <1, i.e., suppressed growth, correlate prominently with the 30 yr periodicities (Figs. 4a–b). The early Middle Triassic chronologies are more similar to the latest Permian chronologies, with prominent ~30 yr periods, but contain an abundance of subdecadal oscillations (Fig. 3). Moreover, the phase correlations of the early Middle Triassic chronologies are with RWI values >1, i.e., enhanced growth (Figs. 4c–d). The CWT analysis, therefore, provides information about decadal to subdecadal paleoclimate through space and time. The results of this analysis indicate a profound shift in paleoclimate in Antarctica during the Late Permian, and that this climate shift corresponded to decreased wood growth, or stress on Late Permian trees. The CWT analysis further suggests that the geospatial climate gradient decreased over time, beginning in the Late Permian and extending into the early Middle Triassic.

5. Cross-wavelet transform to test replicability of the dendrochronology results

CWT results from two independently measured (by ELG and VC) TRW chronologies from the Triassic Lashly B Formation, Allan Hills (SVL) are compared via the technique of cross-wavelet transform to produce the cross-wavelet power spectrum and wavelet coherence (Torrence and Compo, 1998). This technique is applied here, via the Wavelet Comp package in R, to evaluate how reproducible TRW chronologies are in deep time as the fossil wood for both chronologies was sampled from closely spaced locations on two vertically associated bedding planes in the Lashly Fm. in the Allan Hills. Thus, while minor differences are expected, the closely spaced nature of these samples in the sedimentary strata suggest a similar distribution of TRW signals should result, without the potential to provide meaningful cross-matches. The cross-wavelet power spectrum provides information about covariance at each unit time between two time series, with higher cross-wavelet power indicating a common power between the two time series (Supplemental Figs. 9–10). Whereas the wavelet coherence provides information on the correlation of two time series per unit time, regardless of the power, providing information on how well-correlated two time series are (Supplemental Figs. 11–12). Coherence values approaching 0 indicate poor correlation, and coherence values approaching 1 indicate high correlation at the specified significance level. The phase of the covariance is reported for the cross-wavelet power spectrum and the wavelet coherence to inspect whether a correlation is positive or negative or whether one time series leads the other. The cross-wavelet power spectrum produces significant power (0.05 significance level) for Fourier periods ranging from 14–16 yrs and 20–64 yrs, with negative correlations existing for Fourier periods 14–16 yrs and positive correlations for Fourier periods 16 yr and 32 yr (Supplemental Figure 10). The TRW chronology measured by ELG lags behind the chronology measured by VC for Fourier period 32 yr, and the opposite is true between Fourier periods 40–50 yrs. Wavelet coherence, ranging from 0–1, is significant at the 0.05 level for Fourier periods 3–4 yrs, 10 yr, and 32 yr (Supplemental Figure 11). Average coherence values are better than 0.85 for the higher frequency signals, and

better than 0.95 for the lower frequency signals (Supplemental Figure 12). The phase is complex for higher frequencies, displaying both lag/lead patterns and positive/negative correlations with respect to time. However, the phase is more organized at lower frequencies with either the ELG chronology lagging behind the VC chronology, or positive correlation between the two chronologies. The range of Fourier periods with significant cross-wavelet power and wavelet coherence are identical to the significant Fourier periods identified in each chronology individually using CWT analysis, with 0.95 average wavelet coherence for the prominent lower frequency signals in both TRW chronologies.

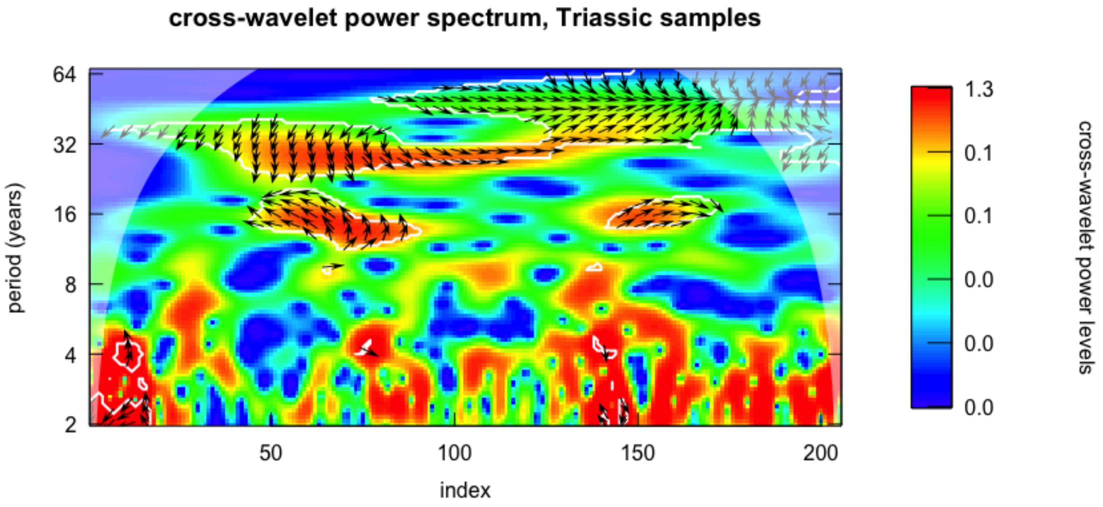

Supplemental Figure 9. Cross-wavelet power spectrum for Triassic RWI chronologies. The heatmap indicates the cross-wavelet power level, with values approaching 1 having more common power between the two chronologies. The arrows indicate information about positive versus negative correlations power (e.g., pointing right or pointing left, respectively). Or for leading/lagging chronologies (pointing up/down, respectively). Arrows pointing along a diagonal or oblique orientation, thus, reflect a combination of the correlation to wavelet power and

leading/lagging chronology.

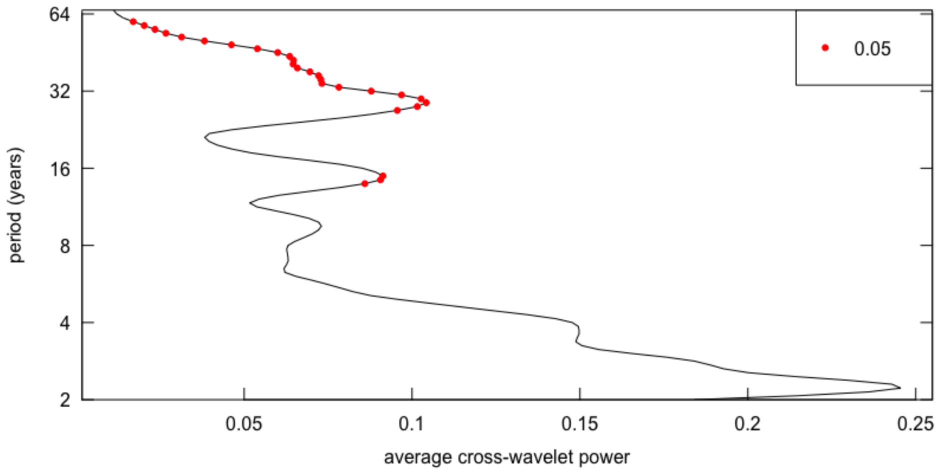

Supplemental Figure 10. Average cross-wavelet power for the Triassic chronologies. The red dots indicate cross-wavelet power at the 0.05 significance level against the null hypothesis of a red noise spectrum.

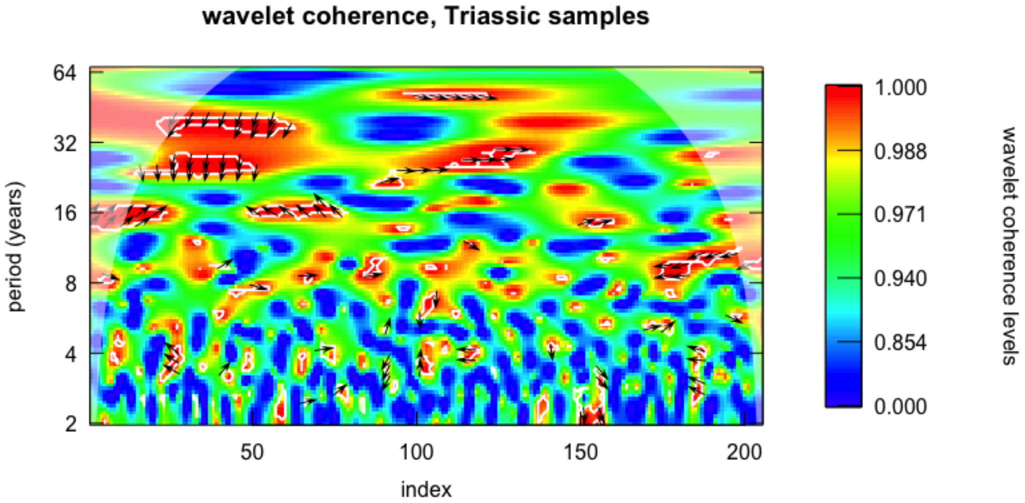

Supplemental Figure 11. Wavelet coherence for the two Triassic chronologies. Arrows possess the same information as in Supplemental Figure 10.

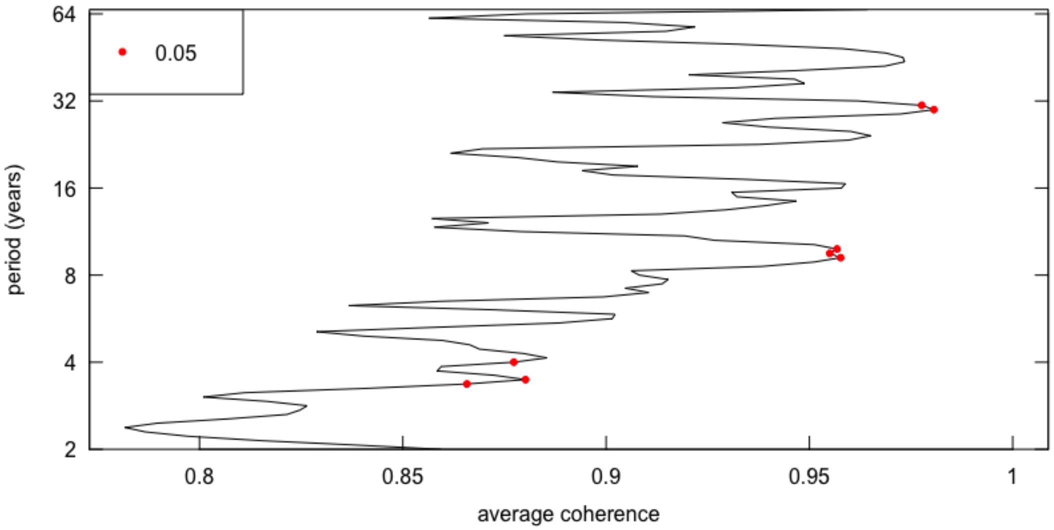

Supplemental Figure 12. Average wavelet coherence for the two Triassic chronologies. The red dots indicate significant (at the 0.05 significance level) wavelet coherence values. The Fourier Period of these significant wavelet coherence values aligns perfectly with the significant periodicities resolved from the CWT analysis of each time series.

6. Paleosol Geochemistry

The studied paleosol was measured, described, and taxonomically classified in the field (Gulbranson et al., 2020). Samples of paleosol material were collected from each horizon, attempting to average the composition of each horizon, by digging inwards ~20 cm into the slope. Subsamples of each horizon were milled into a fine mesh powder and homogenized. 150 mg of sample powder from each sample was digested in a muffle furnace at 1000ºC in the presence of a Li-metaborate flux for 15 minutes and decanted into a polystyrene beaker containing 6 N HCl. The sample+HCl mixture was vigorously shaken for 20 minutes until all of the sample glass had dissolved. The sample+HCl mixture was diluted to 20-fold dilution. Diluted samples were injected into an ICP-MS at Gustavus Adolphus College for the measurement of major element concentrations (Al, Ca, Mg, Na, K). Raw concentrations underwent a standard data reduction procedure based on the results of secondary laboratory references for these elements over a range of concentrations that bracketed the expected results. The corrected major element data is shown in Supplemental Figure 13. By examining specific ensembles of major element concentrations that are known to correlate to soil-forming processes it is evident that this paleosol profile preserves, to high-fidelity, the process of clay accumulation, redoximorphic reactions, and the extent of chemical weathering. Clay accumulation is evidenced principally by Al-content, as Al tends to remain as a residual product in the soil due to chemical weathering of rock-forming minerals. There is a nearly 3-fold increase in Al in the Bt horizons of this paleosol. In addition, the eluviation of Ca ions from the soil solution has been demonstrated to be a

necessary precursor process that promotes illuvial accumulation of clay. The data indicate a nearly 3-fold decrease in Ca concentrations in the Bt horizons relative to the parent material, which indicates establishment of this precursor Ca-eluviation process. Redox reactions, recognized through redoximorphic color patterns and the presence of Mn-nodules are further indicated by the immense concentration of Mn in the parent material relative to the overlying B horizons. The paleosol geochemistry results and paleoclimate model results are supplied in Supplemental file 4.

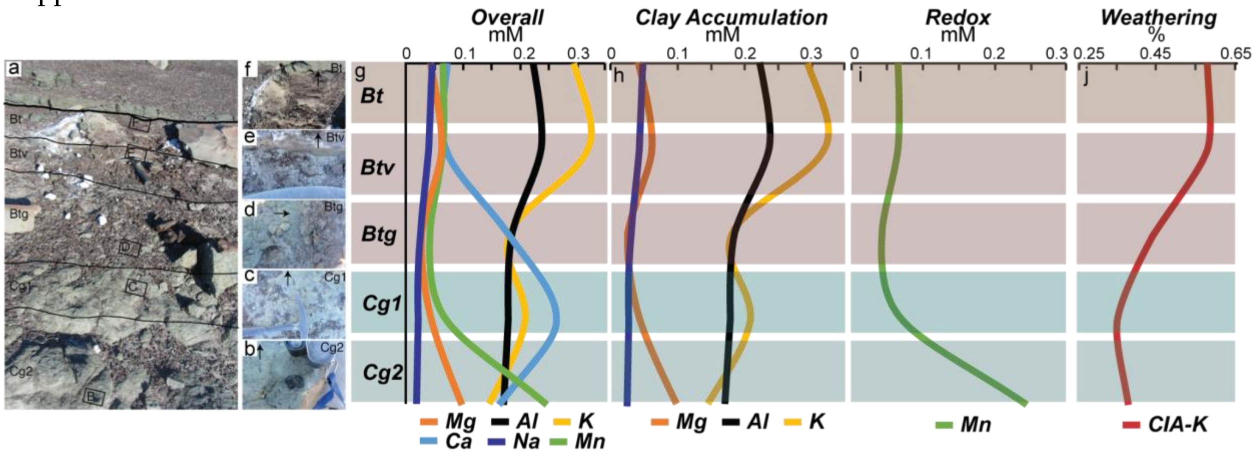

Supplemental Figure 13. Paleosol geochemistry results. a) Paleosol profile, Lashly A Formation, Allan Hills, Classification: gleyed ferritic Argillisol. Images b–f are higher resolution images of each horizon, b) highlights a Mn-nodule (dark object), c) highlights variegated redoximorphic coloration, d) highlights variegated redoximorphic coloration and the occurrence of pedogenic clay accumulation, e) highlights iron nodules in argillic horizon, and f) highlights the argillic horizon and overlying erosional contact with lithic arenite. Visualizations g–j indicate the major element concentrations and extent of chemical weathering. g) the covariance of Na and Ca in the illuvial horizons (Bt–Btg) indicates that Ca eluviation preceded the development of the argillic horizon. h) the development of the argillic horizon is evidenced by clay films in horizons Bt–Btg and by the elevated concentrations of Al, Mg, and K. i) the prevalence of reducing conditions is indicated by the presence of Mn-nodules in b), and by the enhanced Mn concentrations in the Cg2 horizon. j) illustrated the chemical weathering extent via the CIA-K proxy, where the average CIA-K value for the Bt horizons are used in paleoclimate analysis as those values are at least 5% (CIA-K units) greater than the C horizon.
