# Supplementary material for: Paleoclimate-induced stress on polar forested ecosystems prior to the Permian–Triassic mass extinction
Source: Sci Rep. 2022 May 24;12:8702. doi: 10.1038/s41598-022-12842-w (PMC9130125; doi:10.1038/s41598-022-12842-w)
Supplement: Supplementary file 4 — Supplementary Information 4. [file 41598_2022_12842_MOESM4_ESM.html]

SupplementaryFile3.R


# SupplementaryFile3.R

#### erikgulbranson

#### 2022-01-31

```
##header
##Paleoclimate-induced stress on polar forested ecosystems prior to the Permian–Triassic mass extinction

##Erik L. Gulbranson1*, Morgan M. Mellum1, Valentina Corti2, Aidan Dahlseid1, Brian A. Atkinson3, Patricia E. Ryberg4, Gianluca Cornamusini2

##1Department of Geology, Gustavus Adolphus College, 800 W. College Ave, St. Peter, MN 56082 USA
##2Dipartimento di Scienze Fisiche, della Terra e dell’Ambiente, Università di Siena, Italy
##3Department of Ecology and Evolutionary Biology, University of Kansas, 6012 Haworth Hall, Lawrence, KS 66045 USA
##4Department of Physical and Natural Sciences, Park University, Parkville, MO 64152 USA
##*corresponding author Email: erikgulbranson@gustavus.edu

##data entry
data(CR_Dendro_data)
```

```
## Warning in data(CR_Dendro_data): data set 'CR_Dendro_data' not found
```

```
##detrending and indexing
cr.rwi<- detrend(rwl = CR_Dendro_data, method = "Spline")
##chronology and standardization
cr.crn <- chron(cr.rwi, prefix = "CAM", prewhiten = FALSE)
Years<- time(cr.crn)
CAMstd<- cr.crn[, 1]
##wavelet analysis
out.wave <- morlet(y1 = CAMstd, x1 = Years, p2 = 7, dj = 0.1,
                   siglvl = 0.95)
wavelet.plot(out.wave, useRaster = NA, reverse.y = TRUE)
```

```
##sub sample signal strength
cr.sss<- sss(cr.rwi)
cr.ids <- autoread.ids(CR_Dendro_data)
cr.sss2<- sss(cr.rwi,cr.ids)
plot(cr.sss2,type="l",ylim=c(0.2,1),col="darkblue",lwd=2,xlab="Year",ylab="SSS")
lines(cr.sss2,lty="dashed",col="darkgreen",lwd=2)
```

```
##decompose time series into constituent voices using mra in waveslim package
nYrs<- length(Years)
nPwrs2<- trunc(log(nYrs)/log(2))-1
dat.mra<- mra(CAMstd, wf= "la8", J=nPwrs2, method="modwt", boundary="periodic")
YrsLabels<- paste(2^(1:nPwrs2),"yrs", sep="")
plot(Years,rep(1,nYrs),type="n",axes=FALSE,ylab="",xlab="",ylim=c(-3,38))
title(main="Multiresolution decomposition of CAMstd", line=0.75)
axis(side=1)
mtext("Years", side=1, line=1.25)
Offset<-0
dat.mra2<- scale(as.data.frame(dat.mra))
for(i in nPwrs2:1){x<- scale(dat.mra[[i]])+Offset
x<-dat.mra2[,i]+Offset
lines(Years,x)
abline(h=Offset,lty="dashed")
mtext(names(dat.mra)[[i]],side=2,at=Offset,line=0)
mtext(YrsLabels[i],side=4,at=Offset,line=0)
Offset<-Offset+5
}
```
